# Species-Dependent Effects of 3-Hydroxychromone Derivatives on Biofilm Biomass and Planktonic Growth

**DOI:** 10.3390/microorganisms14102295

**Published:** 2026-10-09

**Authors:** Safi Dapetel Balkissou Wouna, Naga Sai Kolli, Kaitlyn Benes, Devin Fauver, Carly L. Salzano, Rohini Parsha, Simran Prajapati, Pier F. Cirillo, Huan Gu

**Affiliations:** Chemistry, Chemical and Biomedical Engineering, University of New Haven, West Haven, CT 06516, USA

**Keywords:** antibiofilm activity, 3-hydroxychromone scaffold, GIC_50_, 50% growth inhibition, *Staphylococcus aureus*, *Pseudomonas aeruginosa*, structure–activity relationship

## Abstract

A series of 3-hydroxychromone derivatives bearing Mannich-type motifs was synthesized and evaluated for effects on biofilm-associated biomass and planktonic growth in *Staphylococcus aureus* ALC2085 and *Pseudomonas aeruginosa* PAO1. Compounds were initially screened at 20 μg/mL using crystal violet staining, followed by experimental 50% growth-inhibitory concentration (GIC_50_) and CFU analyses. Against *S. aureus*, aryl-substituted derivatives had GIC_50_ values >200 μg/mL, whereas 6-bromo derivatives had values of 1.25–10 μg/mL. Thus, the biomass reductions produced by NSK 18 at 20 μg/mL could not be separated from planktonic growth inhibition. Against PAO1, NSK 16 produced statistically significant decreases in biomass, reducing it to 50 ± 24% of the untreated control (*p* = 0.03584), without comparably large or sustained reductions in the measured planktonic-growth endpoints at 20 μg/mL. These findings demonstrate species- and substitution-dependent activity and identify NSK 16 as preliminary PAO1 biomass-reducing hits. Because biomass was measured at a single concentration and not normalized to growth under matched conditions, further concentration-response and viability studies are required to establish biofilm selectivity.

## 1. Introduction

*Staphylococcus aureus* and *Pseudomonas aeruginosa* are clinically important opportunistic pathogens associated with persistent infections and substantial antimicrobial resistance. A systematic global analysis identified both species among the leading bacterial pathogens contributing to deaths associated with antimicrobial resistance in 2019 [1]. *S. aureus* causes infections ranging from skin and soft-tissue infections to pneumonia, endocarditis, osteomyelitis, and bacteremia, whereas *P. aeruginosa* is frequently associated with chronic respiratory infections, wounds, burns, and infections involving implanted medical devices. The clinical management of both organisms is complicated by their ability to form biofilms, which can promote persistence and reduce susceptibility to antimicrobial treatment [2].

Biofilms contain surface-associated bacterial cells and extracellular polymeric substances, including polysaccharides, proteins, extracellular DNA, and other biomolecules [3]. These components create heterogeneous structures that differ physiologically from planktonic cultures and can modify bacterial responses to chemical exposure. For example, the Psl polysaccharide provides rapid protection to developing *P. aeruginosa* biofilms during antibiotic exposure [4]. However, biofilm-associated biomass, bacterial viability, matrix production, initial attachment, and established-biofilm disruption are distinct outcomes. A reduction in one measurement does not necessarily demonstrate a corresponding reduction in the others. Stiefel et al. [5] showed that total-biomass and viable-cell measurements can produce different assessments of biofilm removal, while Skogman et al. [6] demonstrated the value of combining biomass, viability, and matrix measurements when evaluating antimicrobial effects on *S. aureus* biofilms. Therefore, reductions measured using crystal violet staining should be interpreted specifically as changes in retained biofilm-associated biomass rather than direct evidence of bacterial killing, biofilm eradication, or a particular molecular mechanism.

Identifying compounds that reduce biofilm formation without relying primarily on sustained planktonic growth inhibition is of considerable interest. Nevertheless, distinguishing these response types requires direct comparison of biofilm and planktonic-growth measurements. A compound that decreases bacterial proliferation may also reduce the number of cells available to attach, accumulate, and produce matrix. Conversely, a compound may alter attachment or biofilm-associated processes while producing a comparatively smaller effect on planktonic growth. Measurements of biofilm biomass must therefore be interpreted together with growth-inhibition and viable-cell data, particularly when compounds are added at the time of inoculation rather than applied to established biofilms.

Comparative evaluation in *S. aureus* and *P. aeruginosa* can also reveal species-dependent biological activity. These organisms differ in cell-envelope architecture, surface-associated components, biofilm-matrix composition, regulatory networks, and mechanisms governing attachment and community development [2]. Such differences can affect compound uptake and bacterial responses, meaning that activity observed in one species cannot be assumed to predict activity in the other. In this study, the two organisms were used as Gram-positive and Gram-negative laboratory models to compare phenotypic response patterns. The experiments were not designed to determine which specific cellular or molecular differences caused those patterns.

**Figure 1:**
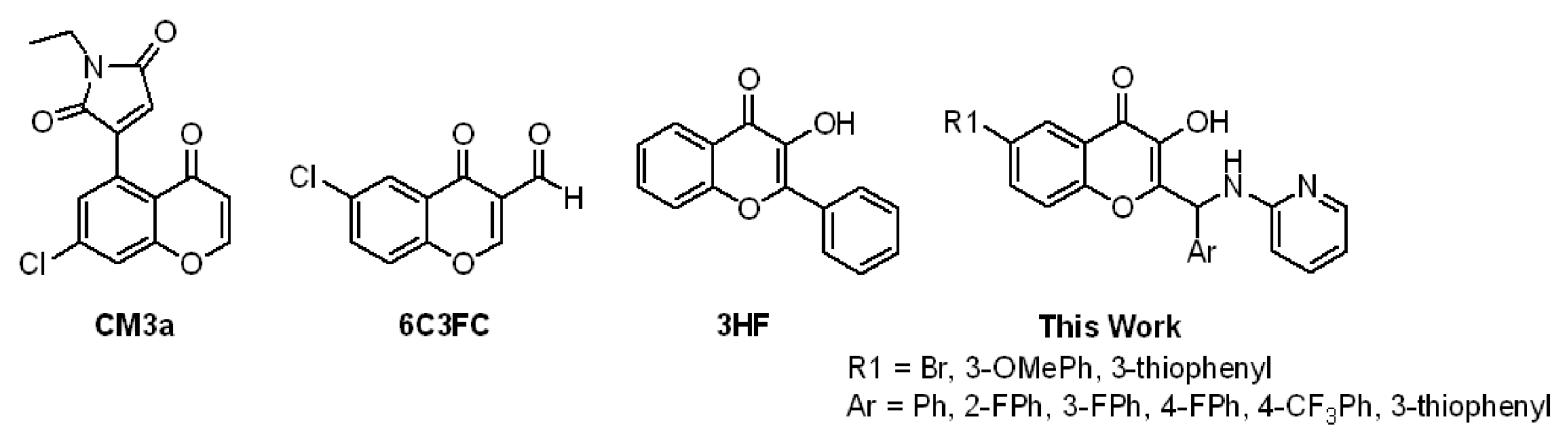
Examples of chromones previously reported in the literature that inhibit biofilm formation and the general 3-hydroxychromone structure investigated in this report.

Chromones are oxygen-containing heterocyclic compounds that provide a chemically versatile framework for structural modification. Previous investigations have reported antibacterial and biofilm-associated activities for several chromone-containing molecules. Zhan et al. [7] reported that the chromone derivative CM3a reduced S. aureus biofilm formation and cellular adherence (Figure 1). Sathiyamoorthi et al. [8] subsequently evaluated chromone and substituted formylchromone derivatives against *Vibrio parahaemolyticus* and *Vibrio harveyi* and identified compounds with antibacterial and antibiofilm-associated activities (see example in Figure 1). Very recently, 3-hydroxychromone (3HF) has been shown to inhibit biofilm formation and enhance antibiotic activity against *Enterococcus faecalis* and *Enterococcus faecium* [9]. Importantly, some active derivatives also inhibited planktonic growth, reinforcing the need to distinguish reduced biofilm biomass from general growth inhibition. Because the compounds examined in these earlier studies differ structurally from the 3-hydroxychromone derivatives investigated in this study, their reported activities do not establish the biological profiles or molecular targets of the series designed in this study.

The 3-hydroxychromone scaffold contains multiple positions at which substituents can be varied, allowing investigation of relationships between chemical structure and biological activity. In the series designed in this study, 6-aryl-substituted and 6-bromo derivatives were synthesized, together with the introduction of a Mannich base at position 2, to examine whether substitution patterns produce different effects on bacterial growth and biofilm-associated biomass (Figure 1). The biological consequences of these structural modifications, particularly whether they differ between Gram-positive and Gram-negative organisms, remain insufficiently understood. Establishing preliminary structure–activity relationships could help distinguish derivatives associated primarily with planktonic growth inhibition from compounds that warrant further evaluation for preferential effects on biofilm formation.

In this study, a series of structurally related 3-hydroxychromone derivatives were synthesized and evaluated for effects on biofilm-associated biomass and planktonic growth in *S. aureus* ALC2085 and *P. aeruginosa* PAO1. The compounds were initially screened for inhibition of biofilm formation at 20 μg/mL using crystal violet staining. An experimentally defined 50% growth-inhibitory concentration (GIC_50_) and colony-forming unit (CFU) growth kinetics were subsequently used to assess whether the observed biomass reductions were accompanied by planktonic growth inhibition. We hypothesized that structural modification of the 3-hydroxychromone scaffold would produce distinct, species-dependent profiles of biofilm biomass reduction and planktonic growth inhibition. By comparing these responses, we sought to identify preliminary structure–activity relationships and prioritize compounds for further investigation. Because biofilm activity was evaluated at a single concentration using a biomass assay, the compounds identified here are considered preliminary screening hits rather than validated biofilm-selective agents. Their cellular targets and mechanisms of action remain to be determined.

## 2. Materials and Methods

### 2.1. Chemical Synthesis

**Figure 2:**
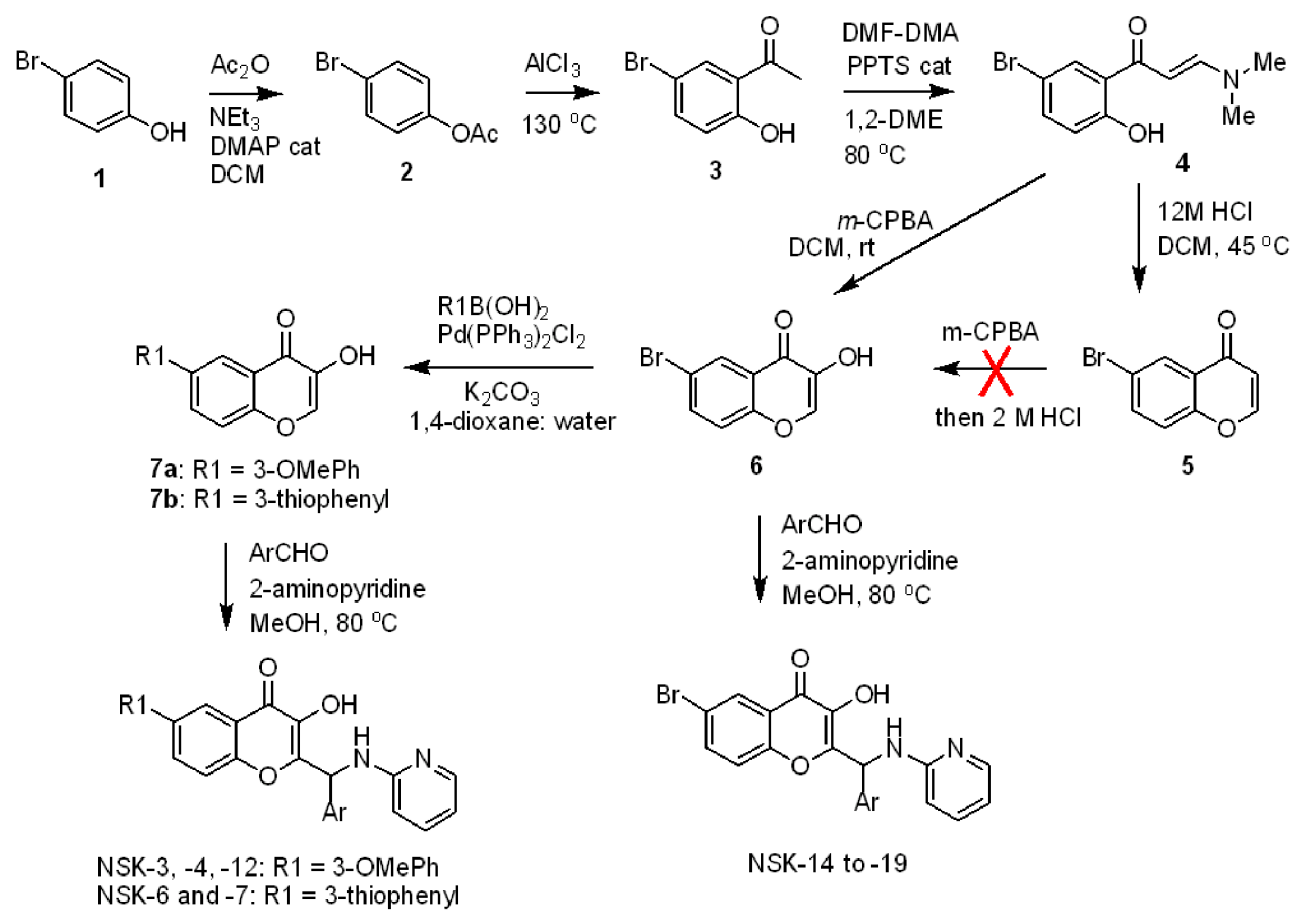
Synthesis of 3-hydroxychromenones described in Table 1.

NSK 3, NSK 4, NSK 6, NSK 7, NSK 12, NSK 14, NSK 15, NSK 16, NSK 18, and NSK-19 were synthesized by following the steps listed in Figure 2 below and Supplementary Information Section S1.

**Table 1:** Chemical structures and molecular information of the compounds synthesized in this study.

Compound Code	R1	Ar	MW (g·mol^−1^)	Molecular Formula
NSK 3	3-OMe–Ph	4-F–Ph	468.48	C_28_H_21_FN_2_O_4_
NSK 4	3-OMe–Ph	4-CF_3_–Ph	518.49	C_29_H_21_F_3_N_2_O_4_
NSK 6	3-Thiophenyl	4-F–Ph	444.48	C_25_H_17_FN_2_O_3_S
NSK 7	3-Thiophenyl	3-Thiophenyl	432.51	C_23_H_16_N_2_O_3_S_2_
NSK 12	3-OMe–Ph	Ph	450.49	C_28_H_22_N_2_O_4_
NSK 14	Br	Ph	423.27	C_21_H_15_BrN_2_O_3_
NSK 15	Br	4-CF_3_–Ph	491.26	C_22_H_14_BrF_3_N_2_O_3_
NSK 16	Br	3-Thiophenyl	429.29	C_19_H_13_BrN_2_O_3_S
NSK 18	Br	3-F–Ph	441.26	C_21_H_14_BrFN_2_O_3_
NSK 19	Br	2-F–Ph	441.26	C_21_H_14_BrFN_2_O_3_

### 2.2. Testing Antibiotics on Bacterial Growth

#### 2.2.1. Bacterial Strains and Culture Conditions

*S. aureus* ALC2085 [10] (SA) and *Pseudomonas aeruginosa* (PAO1, University of Washington) [11] were used in this study. *Staphylococcus aureus* ALC2085 is a specific laboratory strain derived from the *S. aureus* parent strain NCTC8235 (RefSeq Accession Number: NC_007795.1) containing the plasmid pALC2084. *Pseudomonas aeruginosa* PAO1 (PAO1-UW and RefSeq Accession Number: NC_002516) originally arose in Melbourne, Australia (1954) as a chloramphenicol-resistant derivative of the original human wound isolate, PAO1. These strains were chosen to allow for a comparison with other studies [12,13,14,15,16,17]. Frozen stocks were streaked onto BHI (Brain Heart Infusion) agar for SA and Lysogeny Broth (LB) agar for PAO1 (each bacterium was streaked two days before each experiment) and incubated overnight in Lysogeny Broth (LB) at 37 °C without shaking. A well-isolated single colony was selected from each plate for inoculation in 5.0 mL LB and cultured at 37 °C with constant shaking at 200 rpm for exactly 15 h. This protocol ensures recovery of viable cells and establishment of uniform exponential-phase cultures prior to experimental use, as described in standard microbiological practice for biofilm and growth assays [18,19]. Overnight cultures were diluted in fresh LB medium to an optical density at 600 nm (OD_600_) of 0.05 (≈1 × 10^7^ CFU/mL), consistent with previous studies that use early exponential cultures as inoculum for biofilm formation and growth kinetics [18,20].

#### 2.2.2. Compound Handling for Testing

NSK 3, NSK 4, NSK 6, NSK 7, NSK 12, NSK 14, NSK 15, NSK 16, NSK 18, and NSK 19 were dissolved in dimethyl sulfoxide (DMSO) to prepare compound stock solutions. For the initial biofilm screen, 0.5 μL of a 10 mg/mL stock solution was added to each well to produce a final compound concentration of 20 μg/mL in a total assay volume of 249.5 μL. The final DMSO concentration was maintained at 0.2% (*v*/*v*) in all compound-treated and vehicle-control wells. The 20 μg/mL concentration was selected as a practical screening concentration at which the compounds remained soluble and no visible precipitation was observed under the assay conditions.

For planktonic growth-inhibition experiments, compound stocks were prepared at concentrations appropriate for the final test series of [200, 100, 80, 40, 20, 10, 5, 2.5, 1, 1.25, or 0.625 μg/mL]. The stock concentrations were prepared at 100, 40, 20, 10, 5, 2.5, 1.25, 0.5, 0.625, or 0.313 mg/mL to make sure that 0.5 μL of compound stock was added to each 249.5 μL assay, corresponding to a final concentration of 0.2% (*v*/*v*). Thus, the solvent concentration was identical across each dilution series. The compound concentrations, stock concentrations, and dilution series used for each organism and compound are provided in Supplementary Table S1.

Untreated-control wells contained bacteria in LB medium without compound or DMSO. Vehicle-control wells contained bacteria in LB medium with 0.2% (*v*/*v*) DMSO but no compound. The vehicle control was compared with the untreated control to determine whether 0.2% DMSO affected planktonic growth or biofilm-associated biomass. Compound-only blank wells containing LB medium, DMSO, and the corresponding compound concentration without bacteria were used to correct for compound-associated absorbance at the optical density at the wavelength of 600 nm (OD_600_) using a microplate reader.

#### 2.2.3. Biofilm Assay in 96 Well Microplates

Biofilm formation assays were conducted in sterile, flat-bottom, 96-well polystyrene microplates using *S. aureus* ALC2085 and *P. aeruginosa* PAO1. Static microtiter-plate assays permit comparative measurement of surface-associated biomass under controlled conditions [18,21].

To inoculate the bacterial solutions for biofilm growth, the OD_600_ of the bacterial overnight cultures was measured using a spectrophotometer. Then, the bacterial overnight cultures were used to inoculate sterile LB medium to an OD_600_ of 0.05, corresponding to approximately 1 × 10^7^ CFU/mL.

Each compound-treated well contained 249.5 μL of bacterial solution and 0.5 μL of compound solution, providing a final assay volume of 250 μL and a compound concentration of 20 μg/mL. The final DMSO concentration was 0.2% (*v*/*v*). Vehicle-control wells contained 249.5 μL of bacterial inoculum and 0.5 μL of DMSO without compound. Untreated biofilm-control wells contained 249.5 μL of bacterial inoculum without compound or DMSO. Sterility and background-control wells contained LB medium without bacteria. Solvent-background wells contained LB medium and 0.2% DMSO without bacteria. Wells treated with 20 μg/mL Ciprofloxacin (CIP) served as positive controls for inhibition of biofilm formation.

The outer wells of each plate were filled with 250 μL of sterile deionized water to reduce evaporation and edge effects [18,21]. Plates inoculated with *P. aeruginosa* PAO1 were incubated statically at 37 °C for 24 h, whereas plates inoculated with *S. aureus* ALC2085 were incubated statically at 37 °C for 48 h. Each condition was tested using three technical replicate wells in each of three independent biological experiments.

#### 2.2.4. Crystal Violet Staining and Biofilm Quantification

Following biofilm incubation, the liquid cultures containing planktonic and loosely associated cells were removed by gently inverting the microplates. The plates were then washed three times by carefully immersing them in sterile deionized water to remove residual planktonic and loosely attached cells without disrupting the retained surface-associated biomass.

Each well was subsequently filled with 250 μL of 0.1% (*w*/*v*) crystal violet solution (Sigma-Aldrich, St. Louis, MO, USA). Crystal violet stains bacterial cells and extracellular-matrix material retained on the well surface and therefore provides a measure of total surface-associated biomass rather than biofilm-cell viability [18,20]. The plates were incubated statically at 37 °C for 15 min. The crystal violet solution was then discarded, and the plates were washed three times with fresh deionized water to remove unbound dye. After washing, the plates were air-dried completely at room temperature overnight.

The retained crystal violet was solubilized by adding 125 μL of 30% (*v*/*v*) acetic acid to each well. The plates were incubated for 15 min at room temperature without shaking, and absorbance was measured at 550 nm using a microplate reader.

For each experimental condition, matched bacteria-free wells containing LB medium, DMSO, and the corresponding compound concentration were subjected to the same incubation, staining, washing, drying, and solubilization procedures. The OD_550_ value from the corresponding bacteria-free condition was subtracted from each experimental measurement to correct for background staining and potential compound-associated optical interference.

Background-corrected biomass values were expressed relative to the DMSO vehicle control, which was defined as 100%. The untreated bacterial control, containing bacteria without compound or DMSO, was analyzed separately to determine whether 0.2% (*v*/*v*) DMSO affected biomass accumulation. Each condition was evaluated in three technical replicate wells in each of three independent biological experiments. Individual biological-replicate values are presented in the figures. Statistical comparisons were performed separately for each bacterial species using One-Way ANOVA analysis of variance followed by Tukey’s multiple-comparison test. Exact *p*-values, effect sizes, and average ± standard deviations are reported with the corresponding results.

Because crystal violet staining measures total retained biomass, these data do not distinguish among reduced cellular attachment, reduced bacterial growth, altered extracellular-matrix production, increased detachment, or changes in cell viability. Accordingly, the results were interpreted together with planktonic-growth and CFU measurements and are described as changes in surface-associated biomass.

#### 2.2.5. Planktonic Growth-Kinetic Assay and Experimental Determination of the 50% Planktonic Growth-Inhibitory Concentration

The concentration producing 50% inhibition of planktonic growth was determined using an experimental broth microdilution assay. This assay was not a standardized clinical MIC assay and was not CLSI-compliant because LB medium, an OD-based inoculum, and a 50% growth-inhibition endpoint were used. Accordingly, the resulting value is designated as an experimental GIC_50_ rather than a conventional MIC.

Overnight cultures of *S. aureus* ALC2085 and *P. aeruginosa* PAO1 were diluted in fresh LB medium to an initial OD_600_ of 0.05. The bacterial suspensions were exposed to the indicated concentrations of each NSK compound in sterile, flat-bottom, 96-well microplates. The compound concentrations, stock concentrations, and dilution series used for each organism and compound are provided in Supplementary Table S1. Adjacent concentrations were prepared using a twofold dilution factor. The maximum tested concentrations (200 μg/mL for the compounds NSK 3, 4, 6, 7, 12, and 14 and 40 μg/mL for the compounds NSK 15, 16, 18, and 19) were selected based on the limited stock concentration and compound solubility. The final assay volume was 250 μL per well, and the DMSO concentration was maintained at 0.2% (*v*/*v*) in all compound-treated and vehicle-control wells, as described in Section 2.2.2 and Supplementary Table S1.

The plates were incubated at 37 °C with orbital shaking at 200 rpm, and OD_600_ was measured every 30 min for 6 h. Untreated growth-control wells contained bacteria in LB medium without compound or DMSO. Vehicle-control wells contained bacteria in LB medium with 0.2% DMSO but no compound. OD_600_ of the wells with LB, bacteria, and compounds right after mixing and before incubation (t_0_ = 0 min) was used as the background of the corresponding compound and subtracted from the absorbance of the compound-treated bacterial culture at different time points. CIP (concentrations listed in Supplementary Table S1) was included as the antibacterial positive control. Each condition was evaluated in three technical replicate wells in each of three independent biological experiments. Individual biological-replicate values and measures of variability are presented with the corresponding results.

Concentration–response data were analyzed by nonlinear regression using a four-parameter logistic model with log-transformed compound concentration. The experimental GIC_50_ was defined as the fitted concentration producing 50% inhibition relative to the vehicle control. GIC_50_ values are reported with 95% confidence intervals. When 50% inhibition was not reached within the tested range, the result was reported as greater than the highest tested concentration rather than extrapolated beyond the experimental data. Each assay included three technical replicate wells and three independent biological experiments. Individual biological-replicate values are provided in the Data Repository Figshare (https://doi.org/10.6084/m9.figshare.33296406).

#### 2.2.6. Colony-Forming Unit Counts for Validation of Planktonic Growth

Colony-forming unit (CFU) enumeration was used to determine whether exposure to the synthesized 3-hydroxychromone derivatives affected viable planktonic-cell counts [22]. Overnight cultures of *S. aureus* ALC2085 and *P. aeruginosa* PAO1 were prepared as described in Section 2.2.1 and diluted in LB medium to an initial OD_600_ of 0.05. Each culture contained 250 μL and was exposed to compounds with concentrations listed in Supplementary Information Table S1. Untreated cultures and vehicle controls containing 0.2% (*v*/*v*) DMSO were analyzed in parallel.

After compound addition, 20 µL was collected from each culture every 30 min. Each sample was serially diluted in 0.85% NaCl using 10-fold dilutions over the range of 1.0 × 10^2^–1.0 × 10^10^. For each selected dilution, 10 µL was spread onto LB agar. Each dilution was plated in duplicate, and plates were incubated at 37 °C for 15 h. Plates containing 10–50 colonies were used to calculate viable-cell concentrations, which were reported as CFU/mL. The assay’s limit of detection was 200 CFU/mL, calculated from the plated volume and lowest dilution.

The experiment included three independent biological replicates, with two technical plating replicates per biological replicate. Technical replicate counts were averaged to obtain one CFU/mL value for each biological replicate. CFU/mL values were log_10_-transformed before statistical analysis. Log_10_-transformed CFU/mL values were analyzed using two-way ANOVA, with treatment and time as factors, followed by Dunnett’s multiple-comparisons test to compare each treatment with the vehicle control at each time point. Statistical significance was defined as *p* < 0.05.

CFU results were interpreted together with the OD_600_ growth curves and the experimental 50% growth-inhibitory concentration. Because CFU enumeration measures culturable viable cells, it was used to determine whether changes in optical density or attached biomass were accompanied by transient or sustained changes in viable planktonic-cell numbers.

## 3. Results

### 3.1. Chemical Synthesis and Structural Validation

The compounds were synthesized through the multistep route shown in Figure 2. Acylation of 4-bromophenol **1**, followed by a Fries rearrangement, furnished acetophenone **3**. Reaction with dimethylformamide dimethylacetal (DMF-DMA) afforded enaminone **4**. Direct epoxidation of enaminone **4**, as described by Gudipati et al. [23], furnished compound **6** in good yield. In contrast, conversion of **5** to **6** using m-CPBA, as described by Prasad Rao and Srimanarayana [24], did not provide sufficient yield or purity in our hands, even with large excesses of m-CPBA. Compound **6** was subjected directly to a Mannich reaction or first coupled to an arylboronic acid through a Suzuki reaction to form intermediate **7**. The compounds evaluated in this study are listed in Table 1 and coded NSK 3 through NSK 19.

Overall yields varied depending on substitution pattern, with epoxidation identified as the lowest-yielding step. Structural confirmation of all compounds was achieved through standard spectroscopic characterization, and molecular information is provided in Supplementary Information Section S2 and summarized in Table 1. The resulting series comprised Suzuki-coupled 6-aryl derivatives and derivatives retaining the 6-bromo substituent, with additional variation in side-chain structure at position 2. This focused compound set enabled preliminary evaluation of how structural variation influenced biofilm-associated biomass and planktonic growth in *S. aureus* and *P. aeruginosa* PAO1. Because molecular-target studies were not performed, the observed biological effects are not attributed to a specific target or mechanism.

### 3.2. Initial Antibiofilm Screening Against S. Aureus at 20 μg/mL

**Figure 3:**
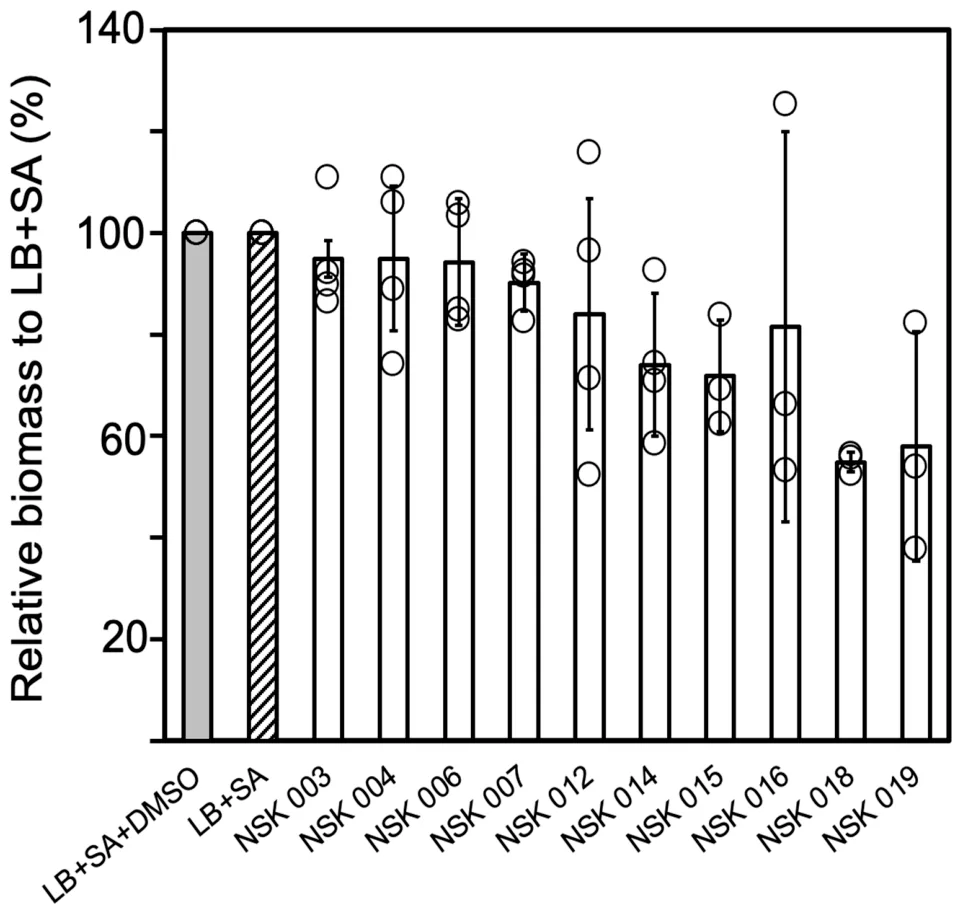
Effect of NSK compounds on *S. aureus* ALC2085 biofilm formation. Biofilm biomass was quantified by crystal violet staining and expressed relative to the untreated control (LB + bacteria, 100%). Data represent mean ± standard deviation from at least three independent experiments. Each condition was evaluated in three technical replicate wells in each of three independent biological experiments.

The synthesized 3-hydroxychromone derivatives were initially screened for antibiofilm activity against *S. aureus* ALC2085 at a fixed concentration of 20 μg/mL using crystal violet staining (Figure 3). This concentration was selected as a practical screening condition based on compound solubility and assay compatibility. The purpose of this first-stage experiment was to identify compounds capable of altering biofilm biomass; the relationship between the screening concentration and planktonic growth inhibition was evaluated subsequently using GIC_50_ and CFU measurements.

**Figure 4:**
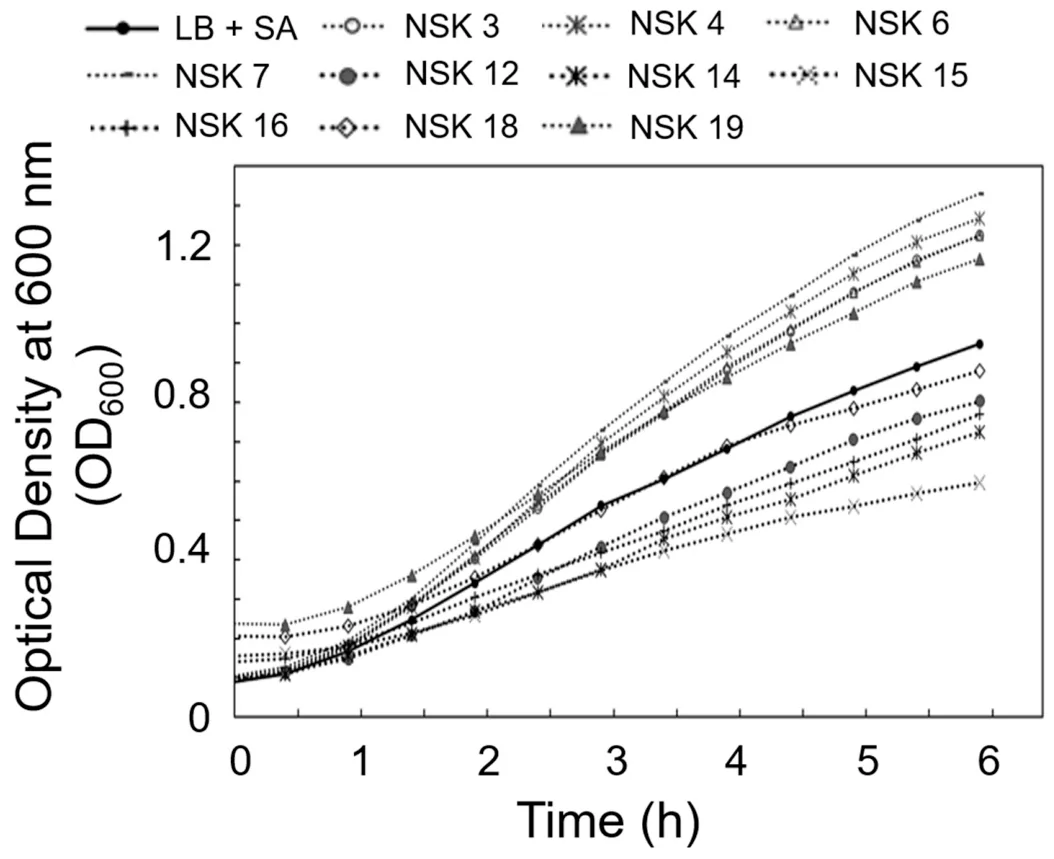
Planktonic growth of *S. aureus* ALC2085 cells in LB medium in the presence of NSK compounds (20 μg/mL). Growth was monitored by OD_600_ over time. The untreated culture served as the growth control. Data represent mean OD_600_ values from at least three independent biological experiments. Each condition was evaluated in three technical replicate wells in each of three independent biological experiments.

Several compounds reduced *S. aureus* ALC2085 biofilm biomass. NSK 18 reduced biomass to 55 ± 2% of the untreated control (*p* = 0.04653), while NSK 19 reduced biomass to 58 ± 22% (*p* = 0.08141) (Figure 3). These results identified NSK 18 as the statistically significant hit in the fixed-concentration *S. aureus ALC2085* biofilm screen. The planktonic *S. aureus* ALC2085 growth test with 20 μg/mL NSK compounds (Figure 4) shows that NSK 18 did not inhibit *S. aureus* growth and NSK-19 promoted cell growth at 6 h after inoculation. Because this experiment alone does not distinguish selective interference with biofilm formation from reduced bacterial growth, the hits were further interpreted together with the subsequent GIC_50_ and CFU analyses described in Section 3.4.

### 3.3. Initial Antibiofilm Screening Against P. Aeruginosa at 20 μg/mL

**Figure 5:**
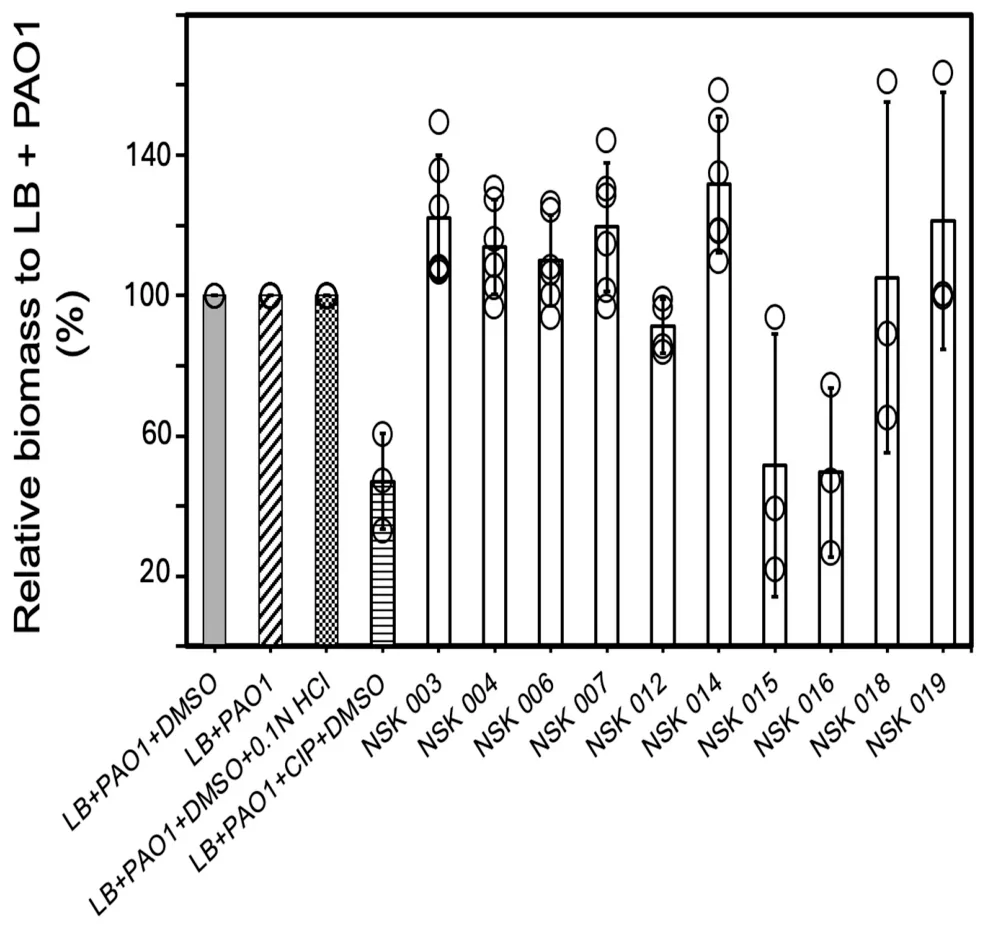
Effect of NSK compounds on *P. aeruginosa* PAO1 biofilm formation. Biofilm biomass was quantified by crystal violet staining and expressed relative to the untreated control (LB + bacteria, 100%). Data represent mean ± standard deviation from at least three independent experiments.

The same fixed-concentration screen was then applied to *Pseudomonas aeruginosa* PAO1 to determine whether the chromone scaffold could alter biofilm formation in a Gram-negative organism (Figure 5). As in the *S. aureus* experiment, 20 μg/mL was used as the initial screening concentration based on compound handling and solubility. This experiment therefore provided an independent phenotypic screen before the contribution of planktonic growth inhibition was assessed.

Several derivatives altered *P. aeruginosa* PAO1 biofilm-associated biomass when tested at 20 μg/mL (Figure 5). NSK 15 and NSK 16 produced the largest decreases, reducing relative biomass to 52 ± 37% and 50 ± 24% of the untreated biofilm control (*p* = 0.05389 and 0.03584), respectively. This reduction is very similar to that of a clinically available antibiotic, CIP, at the same concentration (Figure 5). In contrast, other derivatives produced smaller decreases, no detectable change, or increases in attached biomass. These results indicate that the effect on biofilm-associated biomass varied across the compound series rather than representing a uniform property of the 3-hydroxychromone scaffold.

**Figure 6:**
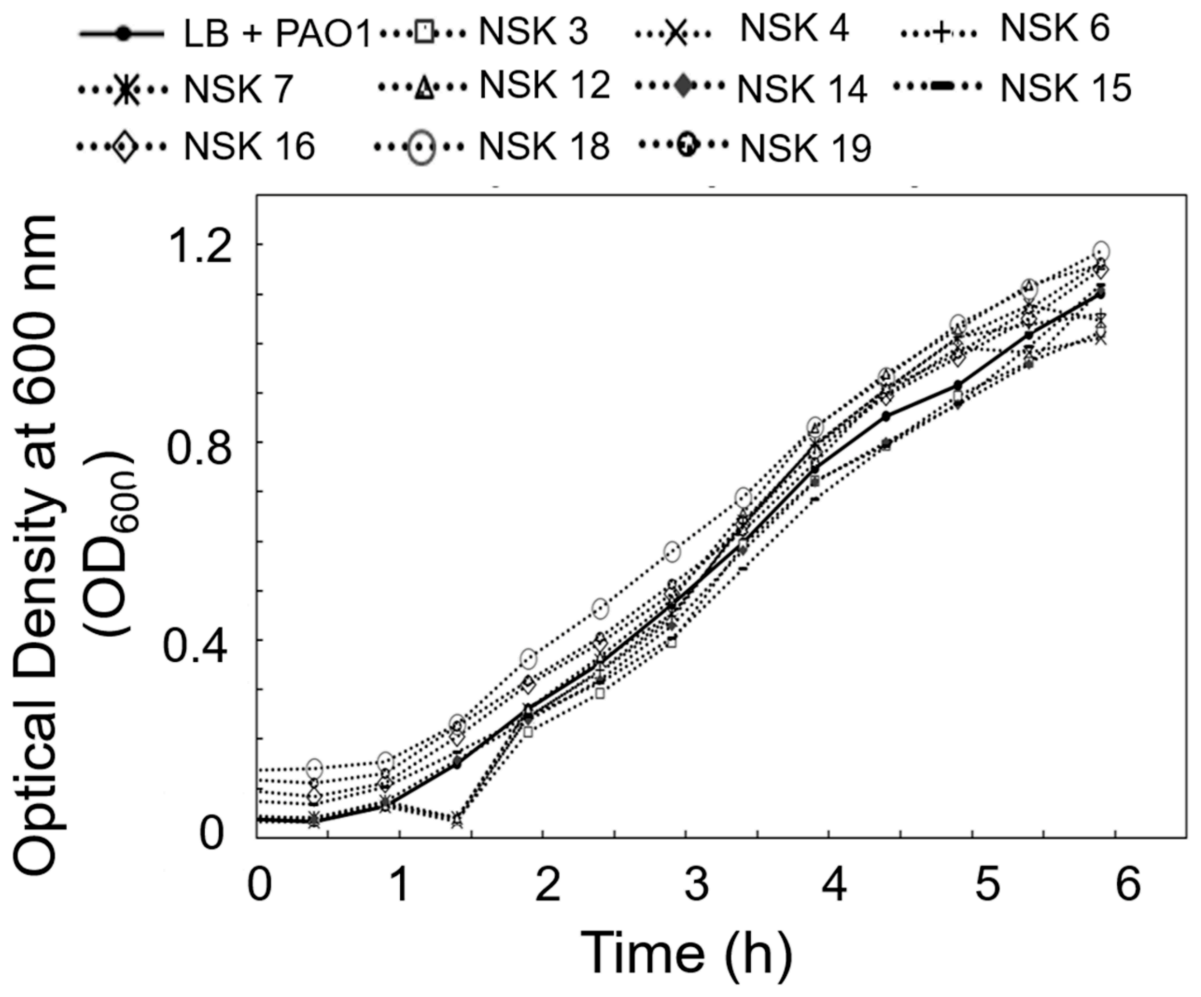
Planktonic growth of PAO1 in LB medium in the presence of NSK compounds (20 μg/mL). Growth was monitored by measuring OD_600_ over time. The untreated culture (LB + PAO1) served as the positive control. Data represent mean OD_600_ values from at least three independent experiments.

During the separate 6 h planktonic-growth experiment, PAO1 cultures exposed to 20 μg/mL of the NSK compounds exhibited broadly similar OD_600_ growth trajectories, and neither NSK 15 nor NSK 16 caused pronounced or sustained suppression of optical density relative to the untreated control (Figure 6). Thus, at the screening concentration, the approximately 50% reductions in crystal violet-stained biomass produced by NSK 15 and NSK 16 were not accompanied by comparably large changes in planktonic optical density during the first 6 h. However, because the planktonic-growth and biofilm-biomass measurements covered different incubation periods, these results alone cannot establish biofilm-selective activity. The findings were therefore interpreted together with the longer-term concentration–response and CFU measurements. The molecular targets and mechanisms underlying the observed effects were not determined.

### 3.4. Experimental 50% Growth-Inhibitory Concentrations and CFU Kinetics

**Figure 7:**
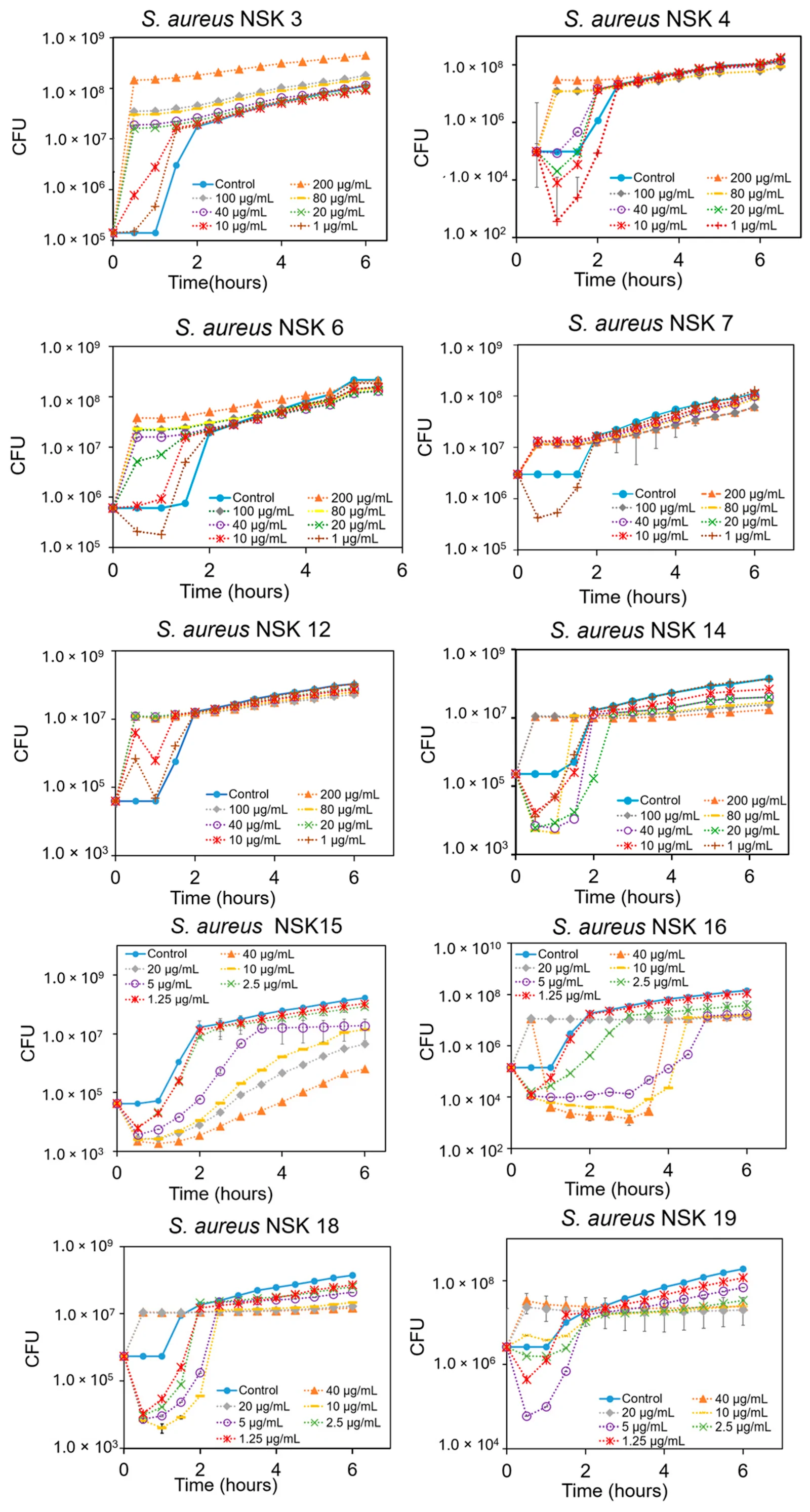
CFU growth kinetics of *S. aureus* ALC2085 exposed to NSK compounds over the indicated concentration ranges. Viable-cell counts were followed for 6 h and are presented on logarithmic scales. The untreated culture served as the control (*n* = 3).

**Figure 8:**
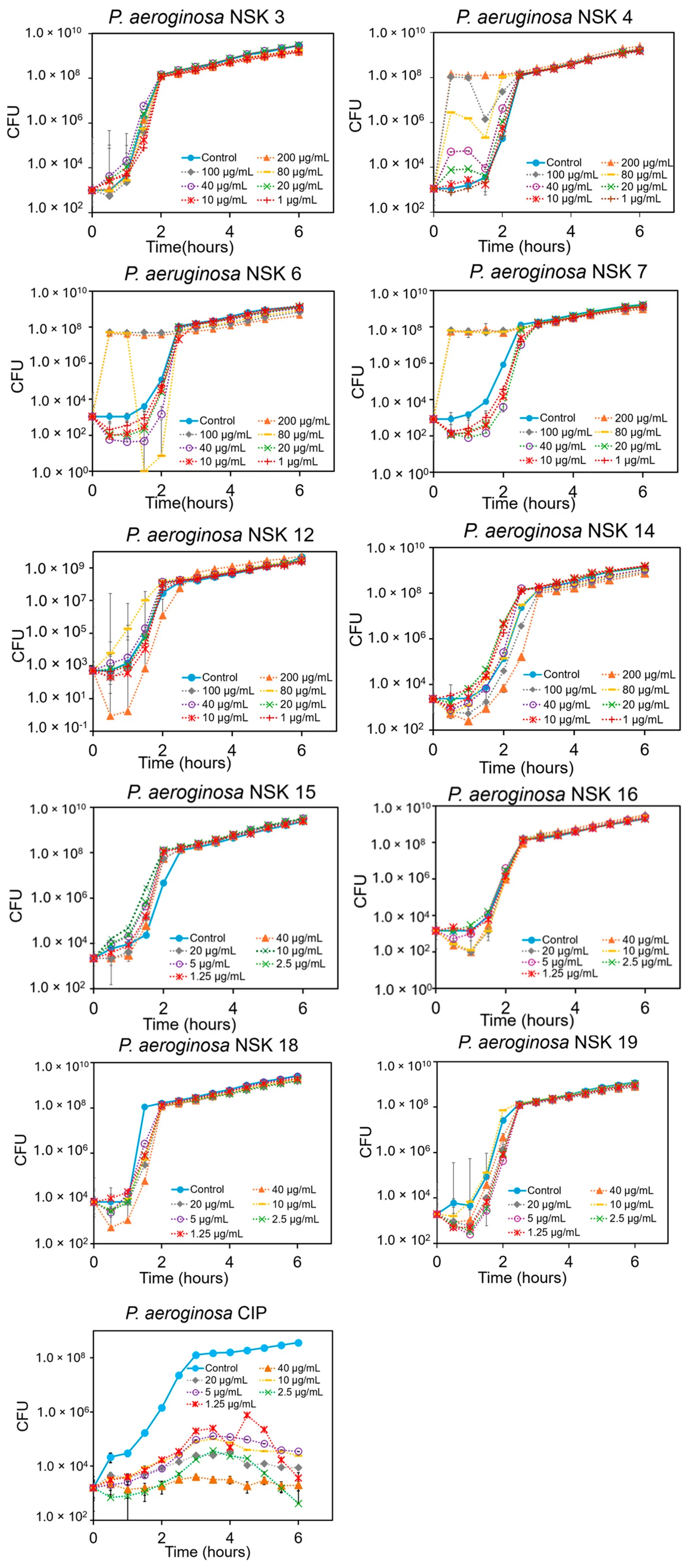
CFU growth kinetics of *P. aeruginosa* PAO1 exposed to NSK compounds over the indicated concentration ranges. Viable-cell counts were followed for 6 h and are presented on logarithmic scales. The untreated culture served as the control (*n* = 3).

Following the fixed-concentration biofilm-biomass screen, endpoint planktonic-growth and CFU measurements were used to assess whether the effects observed at 20 μg/mL were accompanied by inhibition of planktonic growth. The experimental 50% growth-inhibitory concentration (GIC_50_) was defined as the lowest tested concentration that reduced endpoint OD_600_ by at least 50% relative to the vehicle control. This experimental endpoint is not equivalent to a conventional clinical MIC or a fitted IC_50_. The GIC_50_ values are reported in Table 2, while the 6 h CFU profiles show the timing and persistence of changes in culturable planktonic-cell numbers (Figure 7 and Figure 8).

Against *S. aureus* ALC2085, NSK 3, NSK 4, NSK 6, NSK 7, and NSK 12 did not reach 50% endpoint growth inhibition at concentrations up to 200 μg/mL. In contrast, NSK 14, NSK 15, NSK 16, NSK 18, and NSK 19 produced GIC_50_ values of 10, 2.5, 1.25, 1.25, and 1.25 μg/mL, respectively. Thus, the 20 μg/mL concentration used in the biofilm-biomass screen exceeded the GIC_50_ values of all five compounds. The reductions in *S. aureus* biofilm-associated biomass observed for NSK 18 at 20 μg/mL therefore cannot be separated from its effects on planktonic growth.

The *S. aureus* CFU profiles supported this interpretation (Figure 7). NSK 15 and NSK 16 produced concentration-dependent delays or sustained reductions in culturable-cell numbers, particularly at the higher tested concentrations. NSK 14, NSK 18, and NSK 19 also produced early reductions or growth delays, although the magnitude and persistence of these effects varied with concentration. In contrast, cultures exposed to NSK 3, NSK 4, NSK 6, NSK 7, or NSK 12 generally approached the control CFU range by the end of the 6 h experiment, despite some early differences. Collectively, the endpoint and CFU results indicate that the biofilm-biomass effects of the low-GIC_50_ compounds against *S. aureus* should be interpreted as occurring in the presence of measurable planktonic growth inhibition rather than as biofilm-selective effects.

A different pattern was observed for *P. aeruginosa* PAO1. NSK 3, NSK 4, NSK 7, NSK 12, and NSK 14 did not reach 50% endpoint growth inhibition at concentrations up to 200 μg/mL, whereas NSK 6 had a GIC_50_ of 80 μg/mL. For NSK 15, 50% inhibition was not reached at concentrations up to 20 μg/mL, and for NSK 16, NSK 18, and NSK 19, it was not reached at concentrations up to 40 μg/mL. Therefore, none of the compounds produced at least 50% endpoint growth inhibition at the 20 μg/mL concentration used in the PAO1 biofilm-biomass screen. This finding does not demonstrate an absence of growth effects at 20 μg/mL, because concentrations below the GIC_50_ may still cause partial inhibition or changes in growth kinetics. Under our experimental conditions, the GIC_50_ of CIP was found to be smaller than 1.25 μg/mL, which is consistent with the literature [25]. This result suggests that its reduction on PAO1 biofilms could be due to its effects on planktonic growth.

The PAO1 CFU profiles showed transient, concentration-dependent differences during approximately the first 2 h of exposure, but the viable-cell counts generally approached similar ranges by approximately 3–6 h across the tested conditions (Figure 8). In particular, NSK 15 and NSK 16, the compounds producing the largest reductions in PAO1 biofilm-associated biomass at 20 μg/mL, did not produce a comparably large or sustained reduction in CFU during the 6 h planktonic-growth experiment. These findings support their identification as the strongest screening candidates for preferential reduction in PAO1 biofilm-associated biomass. Nevertheless, biofilm-selective activity cannot be established from these experiments alone because the biofilm assay was conducted at a single concentration and over a longer incubation period than the CFU experiment. Matched concentration–response measurements of biofilm-associated biomass and planktonic growth over comparable exposure periods would be required to quantify biofilm selectivity.

## 4. Discussion

This study demonstrates that the biological effects of the synthesized 3-hydroxychromone derivatives depend on both chemical substitution and bacterial species. Integrating crystal violet biomass measurements with endpoint planktonic-growth and CFU data revealed two principal activity profiles. Several 6-bromo derivatives produced substantial planktonic growth inhibition against *S. aureus* ALC2085, whereas NSK 16 reduced *P. aeruginosa* PAO1 biofilm-associated biomass without producing comparably large or sustained changes in the measured planktonic-growth endpoints at the screening concentration. These findings identify the 3-hydroxychromone scaffold as a chemically tunable platform but do not establish biofilm-specific activity or a particular molecular mechanism.

Against *S. aureus* ALC2085, the experimental 50% growth-inhibitory concentration data revealed a pronounced association between substitution pattern and planktonic-growth activity. NSK 3, NSK 4, NSK 6, NSK 7, and NSK 12 did not reach 50% endpoint growth inhibition at concentrations up to 200 μg/mL. In contrast, the 6-bromo derivatives NSK 14, NSK 15, NSK 16, NSK 18, and NSK 19 produced GIC_50_ values ranging from 1.25 to 10 μg/mL. Because the fixed-concentration biomass screen was performed at 20 μg/mL, the screening concentration exceeded the GIC_50_ values of all five 6-bromo derivatives. The reductions in *S. aureus* biofilm-associated biomass observed for NSK 18 therefore occurred under conditions associated with substantial planktonic growth inhibition and cannot be interpreted as selective interference with biofilm formation.

The *S. aureus* CFU profiles support this cautious interpretation. NSK 15 and NSK 16 produced concentration-dependent growth delays or sustained reductions in culturable-cell numbers, particularly at the higher tested concentrations. NSK 14, NSK 18, and NSK 19 also produced early reductions or delays, although their magnitude and persistence varied with concentration. By comparison, cultures treated with the higher-GIC_50_ compounds generally approached the control CFU range by the end of the 6 h experiment despite some early differences. Collectively, these findings suggest that retention of the 6-bromo substituent is associated with greater growth-inhibitory activity against *S. aureus*. Additional experiments will be required to determine whether this association reflects changes in cellular uptake, membrane interactions, chemical reactivity, or engagement of an unidentified cellular target.

The PAO1 results differed substantially from those obtained with *S. aureus* ALC2085. At 20 μg/mL, NSK 16 produced statistically significant decreases in crystal violet-stained biomass, reducing the measured biomass to 50 ± 24% of the untreated control (*p* = 0.03584). NSK 16 did not reach the experimental 50% planktonic-growth-inhibition endpoint at 20 μg/mL against PAO1. Moreover, the 6 h CFU profiles for NSK 16 did not show a reduction in viable-cell density comparable in magnitude to their approximately 50% reductions in biofilm-associated biomass. Although transient concentration-dependent differences were observed during the early portion of the CFU experiments, viable-cell densities generally approached similar ranges after approximately 3–6 h. Thus, the PAO1 data identify NSK 16 as the strongest candidate for preferential reduction in attached biomass within the tested series. Compared with ciprofloxacin (CIP), NSK-016 achieved a similar reduction in PAO1 biofilm biomass at 20 μg/mL. At this concentration, CIP substantially inhibited planktonic growth, whereas NSK-016 did not, identifying NSK-016 as a candidate for preferential antibiofilm activity. However, a concentration below the GIC_50_ is not necessarily a growth-neutral concentration, because partial growth inhibition can occur without reaching the predefined 50% threshold. The different durations of the planktonic-growth and biofilm experiments also prevent a direct conclusion that the biomass reductions occurred independently of growth.

The variation in activity across structurally related compounds is consistent with previous evidence that biological activity within chromone-based series can be strongly influenced by substitution. Zhan et al. [7] reported that a chromone derivative reduced *S. aureus* biofilm development and cell adherence, although that compound and its substitution pattern differed from those evaluated here. Formylchromone derivatives have also demonstrated compound-dependent antimicrobial and biofilm-related activity against *Vibrio* species, with halogenated analogues among the more active structures [8]. These reports support further investigation of chromone derivatives as antimicrobial and biofilm-modulating compounds, but they do not establish that different chromone series act through a shared molecular mechanism. The species-dependent responses observed in this study further indicate that activity cannot be assigned to the chromone core alone. It should be noted that a similar Mannich product involving an aryl aldehyde and 2-aminopyridine, but on kojic acid instead of 3-hydroxychromenone, was previously reported as an irreversible sortase inhibitor in Gram-positive bacteria [26]. Inhibition of sortase may therefore be a potential mechanism by which biofilm formation is prevented in SA. However, besides covalent modification of sortase, other targets or processes such as metal complexation and redox cycling may also be operative, though these compounds are not necessarily Pan-Assay Interference Compounds (PAINS) [27]. Future studies will be directed at elucidating these mechanisms.

Crystal violet stains bacterial cells and extracellular matrix components and does not distinguish viable from nonviable cells [5,18,21]. Hence, the crystal violet results must be interpreted specifically as measurements of total attached biomass. Consequently, a lower signal may result from reduced initial attachment, reduced cell proliferation, altered matrix production, loss of cell viability, increased detachment, or a combination of these effects. Comparisons of biofilm-quantification methods have demonstrated that biomass and viability measurements provide different information and that complementary assays are required to distinguish biofilm removal or altered accumulation from bacterial killing [5,6]. Accordingly, the reduced crystal violet signal in this study does not mean the killing of biofilm-associated cells or disruption of established biofilms.

In this study, GIC_50_ was operationally defined as the lowest tested concentration producing at least a 50% reduction in endpoint OD_600_ relative to the vehicle control, which differs from a conventional MIC [22]. The resulting values are therefore useful for comparing compounds within this experimental system but should not be interpreted as clinical MIC values. CFU enumeration provides complementary information about culturable cells, but the 6 h duration does not capture growth effects that might emerge during the longer biofilm incubation.

Several additional limitations should be acknowledged. First, biofilm-associated biomass was evaluated at only one compound concentration, preventing determination of a biofilm concentration-response relationship or a quantitative biofilm inhibitory concentration. Second, planktonic growth and biomass were assessed over different time intervals. Third, attached-cell viability, metabolic activity, biofilm thickness, extracellular polymeric substances, and initial attachment were not measured. Fourth, the CFU experiments evaluated planktonic cultures rather than viable cells recovered directly from the attached biomass. Finally, molecular targets were not investigated; therefore, the observed phenotypes cannot be assigned to a specific protein, cellular pathway, or chemical mechanism.

Future studies should evaluate NSK-16 across matched concentration ranges while measuring PAO1 planktonic growth and attached biomass over comparable exposure periods. Viable-cell enumeration from the attached population, metabolic-activity measurements, microscopy-based analysis of biofilm coverage and thickness, and quantification of extracellular matrix components would help distinguish reduced attachment or matrix accumulation from loss of viability [28]. Experiments should also distinguish prevention of biofilm formation from treatment of established biofilms. These studies would permit quantitative assessment of the separation between planktonic-growth inhibition and reduction in biofilm-associated biomass and would provide a stronger foundation for mechanistic investigation.

## 5. Conclusions

This study identifies 3-hydroxychromone derivatives as a chemically tunable scaffold with species- and substitution-dependent effects on bacterial growth and biofilm-associated biomass. A fixed-concentration screen at 20 μg/mL was followed by experimental 50% growth-inhibitory concentration (GIC_50_) and CFU analyses to assess whether changes in attached biomass were accompanied by planktonic growth inhibition. In *S. aureus* ALC2085, the principal biomass-reducing compound, NSK 18, was tested at concentrations above its GIC_50_ values. Their effects on attached biomass therefore cannot be separated from their inhibition of planktonic growth. In *P. aeruginosa* PAO1, NSK-16 produced statistically significant decreases in crystal violet-stained biomass, reducing it to 50 ± 24% of the untreated control (*p* = 0.03584). NSK 16 did not produce a comparably large or sustained reduction in the measured PAO1 planktonic-growth endpoints at 20 μg/mL. These compounds therefore represent the strongest candidates in this series for preferential reduction in PAO1 biofilm-associated biomass. However, because biomass was measured at only one concentration and crystal violet does not distinguish among attachment, matrix accumulation, viability, and detachment, biofilm-selective activity cannot yet be concluded. Matched concentration-response studies incorporating attached-cell viability, microscopy, and matrix measurements are needed to establish selectivity and determine the mechanisms underlying these effects.

## Figures and Tables

**Table 2:** Experimental 50% growth-inhibitory concentrations of the synthesized compounds against *S. aureus* ALC2085 and *P. aeruginosa* PAO1. GIC_50_ was operationally defined as the lowest tested concentration producing at least a 50% reduction in endpoint OD_600_ relative to the vehicle control. These values are experimental growth-inhibition endpoints and should not be interpreted as conventional clinical MIC values. A value reported as greater than the highest tested concentration indicates that 50% inhibition was not reached within the compound-specific concentration range.

Microorganism	Compound	GIC_50_ (μg/mL)
*S. aureus* ALC2085	NSK 3	>200
	NSK 4	>200
	NSK 6	>200
	NSK 7	>200
	NSK 12	>200
	NSK 14	10
	NSK 15	2.5
	NSK 16	1.25
	NSK 18	1.25
	NSK 19	1.25
*P. aeruginosa* PAO1	NSK 3	>200
	NSK 4	>200
	NSK 6	80
	NSK 7	>200
	NSK 12	>200
	NSK 14	>200
	NSK 15	>20
	NSK 16	>40
	NSK 18	>40
	NSK 19	>40
	CIP	<1.25

## Data Availability

The original data presented in the study are openly available in the data repository Figshare (https://doi.org/10.6084/m9.figshare.33296406).

## Supplementary Materials

The following supporting information can be downloaded at: https://www.mdpi.com/article/10.3390/microorganisms14102295/s1, Table S1: Summary of compound concentrations, stock concentrations, and dilution series used for each microorganism and compound for growth-inhibition experiments.

## Abbreviations

The following abbreviations are used in this manuscript:

SA	*Staphylococcus aureus*
PAO1	*Pseudomonas aeruginosa*
OD	Optical Density read at a specific wavelength
LB	Lysogeny Broth
