# Supplementary material for: Species-Dependent Effects of 3-Hydroxychromone Derivatives on Biofilm Biomass and Planktonic Growth
Source: Microorganisms. 2026 Oct 9;14(10):2295. doi: 10.3390/microorganisms14102295 (PMC13707711; doi:10.3390/microorganisms14102295)
Supplement: Supplementary file 1 [file microorganisms-14-02295-s001.zip › microorganisms-4545070-supplementary.pdf]

Supplementary Information

Species-Dependent Effects of 3-Hydroxychromone Derivatives on Biofilm Biomass and Planktonic Growth

Safi Dapetel Balkissou Wouna, Naga Sai Kolli, Kaitlyn Benes, Devin Fauver, Carly L. Salzano, Rohini Parsha, Simran Prajapati, Pier F. Cirillo*, and Huan Gu*

Department of Chemistry and Chemical & Biomedical Engineering, University of New Haven, 300 Boston Post Road, West Haven, CT 06516

S1. Supplementary Method.

S1.1 Chemical synthesis

Unless otherwise noted, all reagents and solvents were purchased from commercial sources and used without further purification. TLC plates used for determining reaction progress were plastic sheets precoated with SiO_2 60Fm. Flash column chromatography was performed on silica gel (70-230 mesh). All ^{1}H NMR spectra were recorded at 47 or 400 MHz and all ^{13}C NMR spectra (when available) were recorded at 100 MHz at rt in either DMSO-*d6* or $CDCl_3$.

S1.2 4-Bromophenyl acetate, 2.

In a 500 mL round-bottom flask equipped with magnetic stir bar 4-bromophenol (compound **1**, 10.0 g; 57.8 mmol; 1.00 equiv.) was dissolved in 200 mL dichloromethane. Acetic anhydride (6.6 mL; 69.36 mmol; 1.2 equiv.) and triethylamine (12.1 mL; 86.7 mmol; 1.5 equiv.) were then added via syringe at room temperature. The mixture was left stirring for 1.0 hour at ambient temperature, then poured into a separating funnel containing 1M aqueous HCl. The layers were separated and the aqueous layer was further extracted with dichloromethane. The combined organics were washed with brine and dried over Na_2SO_4, filtered, and the solvent was removed *in vacuo*. The crude product was purified by column chromatography eluting with 30% ethyl acetate in hexanes to furnish 11.38 g of product as a pale yellow oil (52.9 mmol; 91.5% yield), though it can also be used as is, without purification, for the next step. ^{1}H NMR (47 MHz; $CDCl_3$): δ (ppm) 7.5 (d, 2 H, Ar-H); 7.0 (d, 2 H, Ar-H); 2.1 (s, 3 H, CH_3CO).

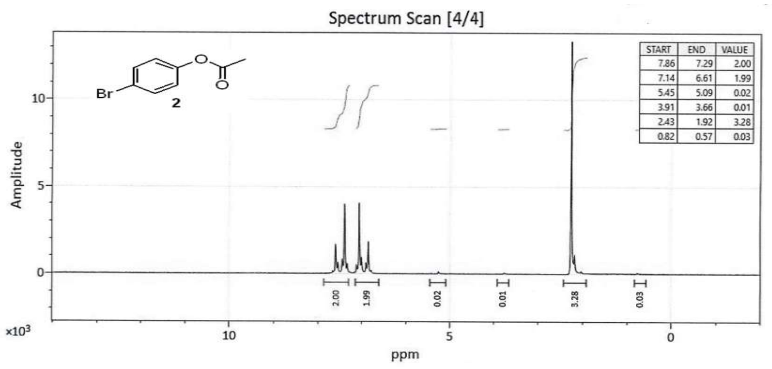

S1.3 5’-bromo-2’-hydroxy-acetophenone, 3.

Acetate ester **2** (11.0 g; 51.2 mmol; 1.0 equiv.) was placed in a 250 mL round-bottom flask equipped with a large magnetic stir bar and treated at room temperature with aluminum chloride (20.48 g; 153.6 mmol; 3.0 equiv.) under argon atmosphere. The mixture was then placed in an oil bath, heated to 130 ºC, and the resulting liquid mixture was stirred for a half hour. It was then cooled to 60-70 ºC and poured into ice water. The product was extracted three times with ethyl acetate. The combined organic layers were washed with brine and dried over Na_2SO_4. The crude product was purified by column chromatography eluting with 45% ethyl acetate in hexanes to afford 8.37 g of desired acetophenone as a yellow solid (38.9 mmol; 76% yield). ^{1}H NMR (400 MHz; $CDCl_3$): δ (ppm) 12.2 (s, 1 H, C2’-OH); 7.96 (d, 1 H, J = 2.5 Hz, C6’H); 7.65 (dd, 1H, J = 2.5 and 5.6 Hz, C4’H); 6.96 (d, 1 H, J = 5.6 Hz, C3’H); 2.64 (s, 3 H, COCH3).

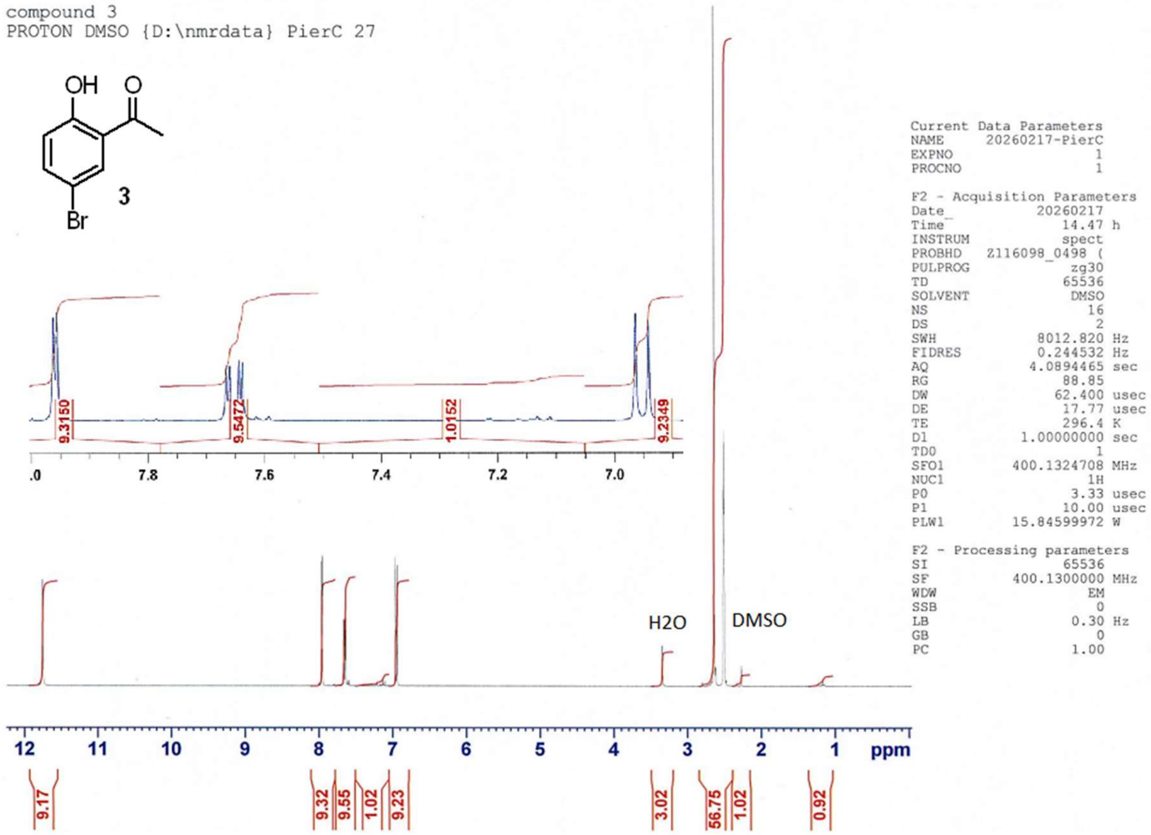

S1.4 1-(5’-bromo-2’-hydroxy-phenyl)-3-*N,N*-dimethylamino-prop-2-en-1-one, 4.

Acetophenone **3** (8.61 g; 40.0 mmol; 1.0 equiv.) was placed in a round-bottom flask and dissolved in 150 mL anhydrous 1,2-dimethoxyethane. Pyridinium *para*-toluenesulfonate was then added as a catalyst (1.14 g; 6.00 mmol; 0.15 equiv.) followed by *N,N*-dimethylformamide dimethylacetal (DMF-DMA) (5.3 mL; 40.0 mmol; 1.0 equiv.). The mixture was heated to reflux overnight, then cooled to ambient temperature. The mixture was poured into water and the precipitate was vacuum filtered on a Buchner funnel. The solid was air dried then placed under high vacuum. 8.72 g of desired enaminone **4** was obtained as a bright orange solid (80.7 % yield). ^{1}H NMR (400 MHz; $SO(CD_3)_2$) δ (ppm)): 14.72 (s, 1 H, OH); 8.12 (d, 1 H, J = 2.4

Hz, C6’H); 7.94 (d, 1 H, J = 11.8 Hz, vinyl H); 7.49 (dd, 1 H, J = 8.8 and 2.4 Hz, C4’H); 6.79 (d, 1 H, J = 8.8 Hz, C3’H); 6.02 (d, 1 H, J = 11.8 Hz, vinyl H); 3.23 (s, 3 H, N-CH3); 3.04 (s, 3 H, N-CH3).

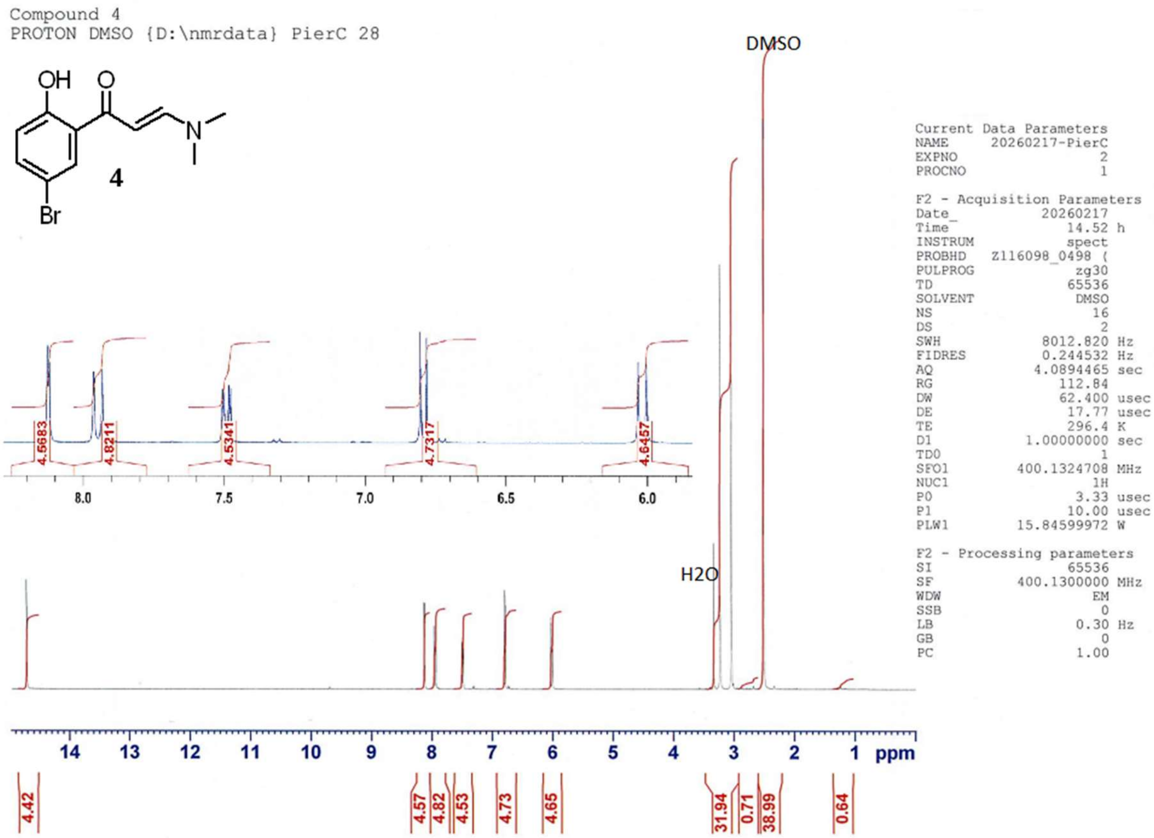

S1.5 6-bromo-3-hydroxy-chromen-4-one, 6.

Crude enaminone **4** (8.72 g; 32.3 mmol; 1 equiv.) was dissolved in 350 mL dichloromethane. Meta-chloroperoxybenzoic acid (m-CPBA; ~70% w/w) (23.9 g; 96.9 mmol; 3.0 equiv.) was then added over 15 minutes, portion wise (NOTE: strong exotherm!!). The orange color of starting material is quickly converted to a pale yellow, and after 2 h a white flocculent precipitate starts to form. The mixture was left stirring vigorously over 36 hours. The mixture was further diluted with dichloromethane, then poured into 100 mL of 1 M aqueous NaOH solution. The layers were separated and the organic layer was sequentially washed with saturated aqueous $NaHCO_3$ and brine. It was then dried (Na_2SO_4), filtered through a short plug of silica gel, which was washed with 40% ethyl acetate in hexanes, and the solvents were removed *in vacuo*. After trituration in hot ethyl acetate and filtration, 6-bromo-3-hydroxychromen-4-one **6** (4.69 g; 60.3% yield) was obtained as a pale yellow solid. (^{1}H NMR (400 MHz; $SO(CD_3)_2$): δ (ppm) 9.35 (br s, 1 H, OH); 8.28 (s, 1 H, H2); 8.18 (d, 1 H, J = 2.4 Hz; H5); 7.91 (dd, 1 H, J = 9.0 and 2.4 Hz; H7); 7.64 (d, 1 H, J = 9.0 Hz; H8). GC-MS m/z = 240 (100%); 242 (97%).

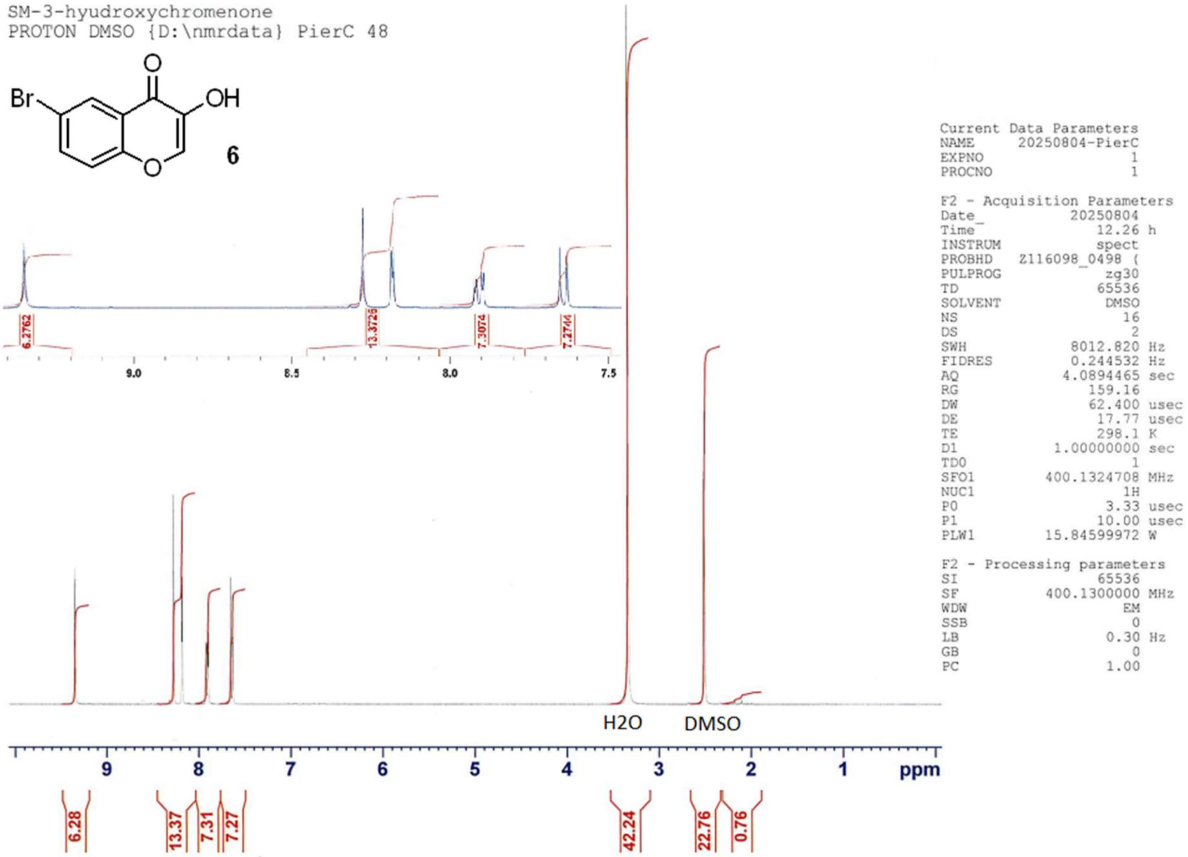
SM-3-hyudroxychromenone
PROTON DMSO {D:\nmrdata} PierC 48
Br
O
OH
O
6
Current Data Parameters
NAME 20250804-PierC
EXPNO 1
PROCNO 1
F2 - Acquisition Parameters
Date_ 20250804
Time 12.26 h
INSTRUM spect
PROBHD Z116098_0498 (
PULPROG zg30
TD 65536
SOLVENT DMSO
NS 16
DS 2
SWH 8012.820 Hz
FIDRES 0.244532 Hz
AQ 4.0894465 sec
RG 159.16
DW 62.400 usec
DE 17.77 usec
TE 298.1 K
D1 1.00000000 sec
TD0 1
SFO1 400.1324708 MHz
NUC1 1H
P0 3.33 usec
P1 10.00 usec
PLW1 15.84599972 W
F2 - Processing parameters
SI 65536
SF 400.1300000 MHz
WDW EM
SSB 0
LB 0.30 Hz
GB 0
PC 1.00
6.2762
13.3726
7.3074
7.2744
9.0
8.5
8.0
7.5
H2O
DMSO
9
8
7
6
5
4
3
2
1
ppm
6.28
13.37
7.31
7.27
42.24
22.76
0.76

C:\Users\User\Desktop\UNH Research\Cirillo Group\NAGA SAI KOLLI\PFC-MK-26.qgd

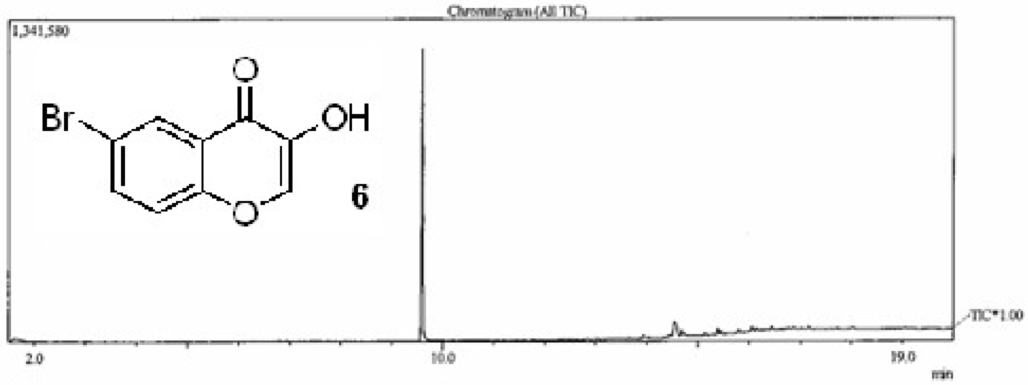

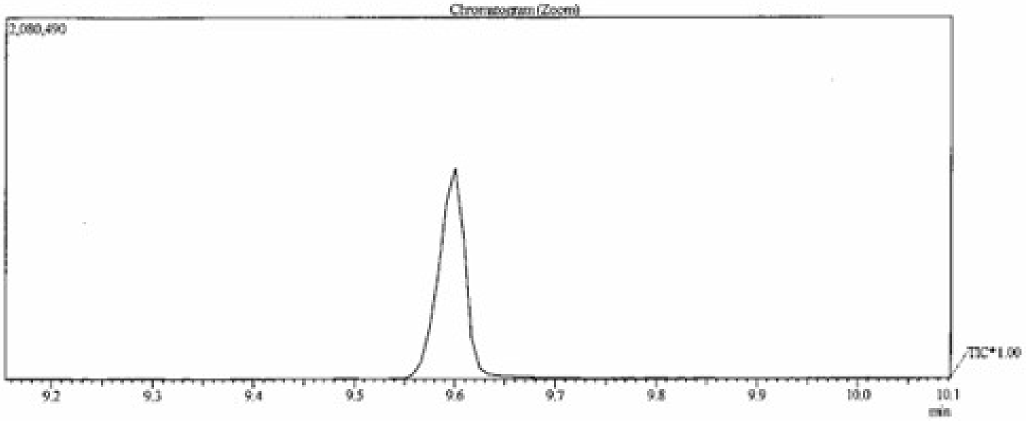

Spectrum

Line#:1 R.Time:9.600(Scan#:973)
MassPeaks:256
RawMode:Single 9.600(973) BasePeak:239.95(149247)
BG Mode:None Group 1 - Event 1 Scan

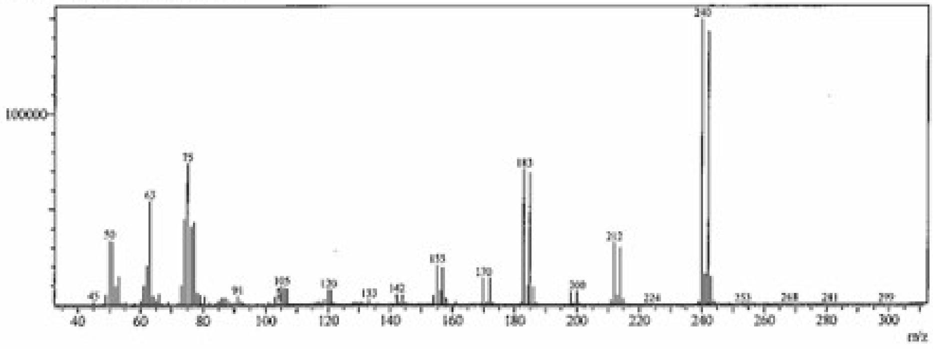

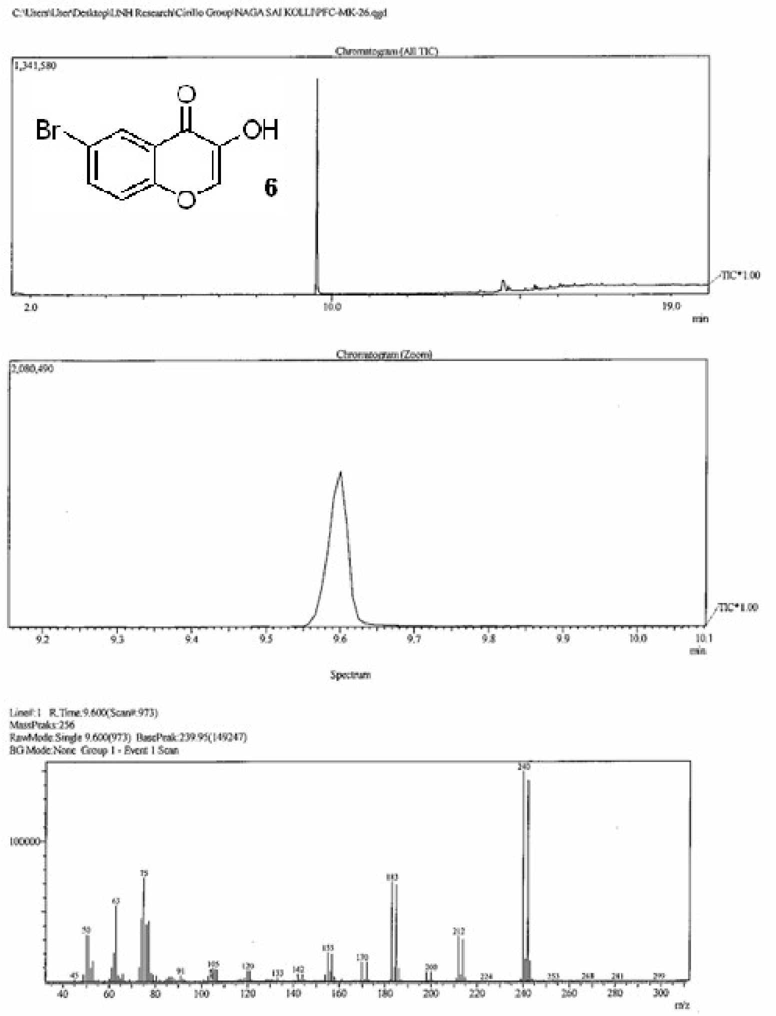

S1.6 6-(3-methoxyphenyl)-3-hydroxy-chromen-4-one, 7a.

Bromo-hydroxy-chromenone **6** (250 mg; 1.04 mmol; 1.0 equiv.) was dissolved in 2.5 mL 1,4-dioxane. Water (1.25 mL) was added, then 3-methoxyphenylboronic acid (188 mg; 1.24 mmol; 1.2 equiv.) and potassium carbonate (286 mg; 2.07 mmol; 2.0 equiv.). The mixture was purged with argon gas for 10 minutes then Pd(dppf)$_2$Cl$_2$ catalyst (37.9 mg; 0.05 mmol; 0.05 equiv.) was added. The mixture was heated at 120 ºC for 2 hours, then it was allowed to cool down and was quenched with water and extracted with ethyl acetate. The combined organic layers were washed with brine, dried with Na_2SO_4, decanted, and the solvent was removed *in vacuo*. The product was purified by column chromatography to afford a tan powder (196 mg; 70% yield). ^{1}H NMR (400 MHz; SO(CD$_3$)$_2$): δ (ppm) 9.22 (s, 1 H, OH); 8.27 (s, 1 H); 8.25 (s, 1 H); 8.05 (d, 1 H); 7.70 (d, 1 H); 7.42 (dd, 1 H); 7.30 (d, 1 H); 7.25 (s, 1 H); 7.00 (d, 1 H); 3.88 (s, 3 H, OCH$_3$). ^{13}C NMR (400 MHz; SO(CD$_3$)$_2$): δ (ppm) 173.2; 160.3; 155.3; 142.4; 141.3; 140.6; 136.9; 132.6; 130.7; 123.4; 122.7; 119.69; 119.66; 114.1; 112.8; 55.70 (OC̲H3).

GC-MS: m/z = 268 (100%).

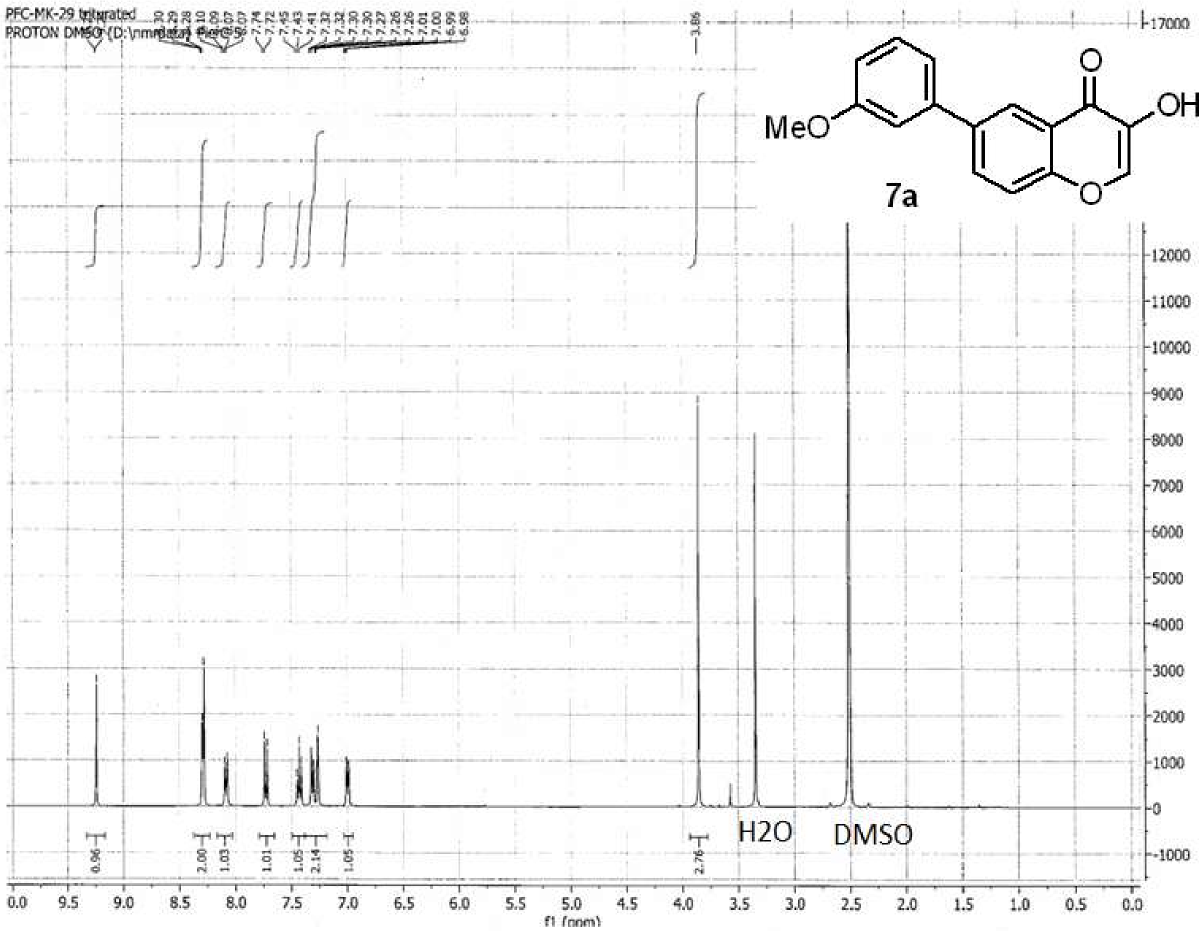
PFC-MK-29 triturated
PROTON DMSO {D:\nmrdata} PierC 5
3.86
MeO
OH
O
7a
H2O
DMSO
0.96
2.00
1.03
1.01
1.05
2.14
1.05
2.76
f1 (ppm)

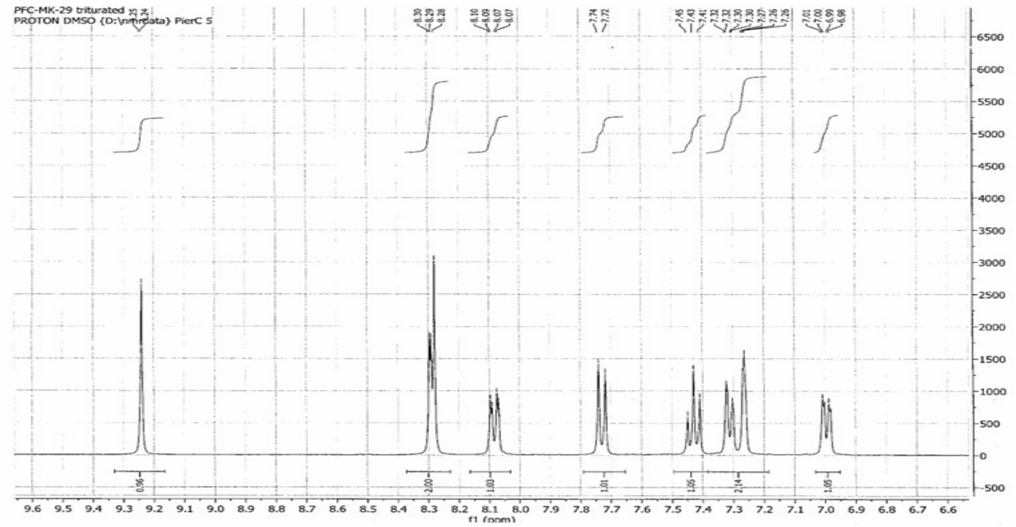
PFC-MK-29 triturated
PROTON DMSO {D:\nmrdata} PierC 5
0.96
2.00
1.03
1.01
1.05
2.14
1.05
f1 (ppm)

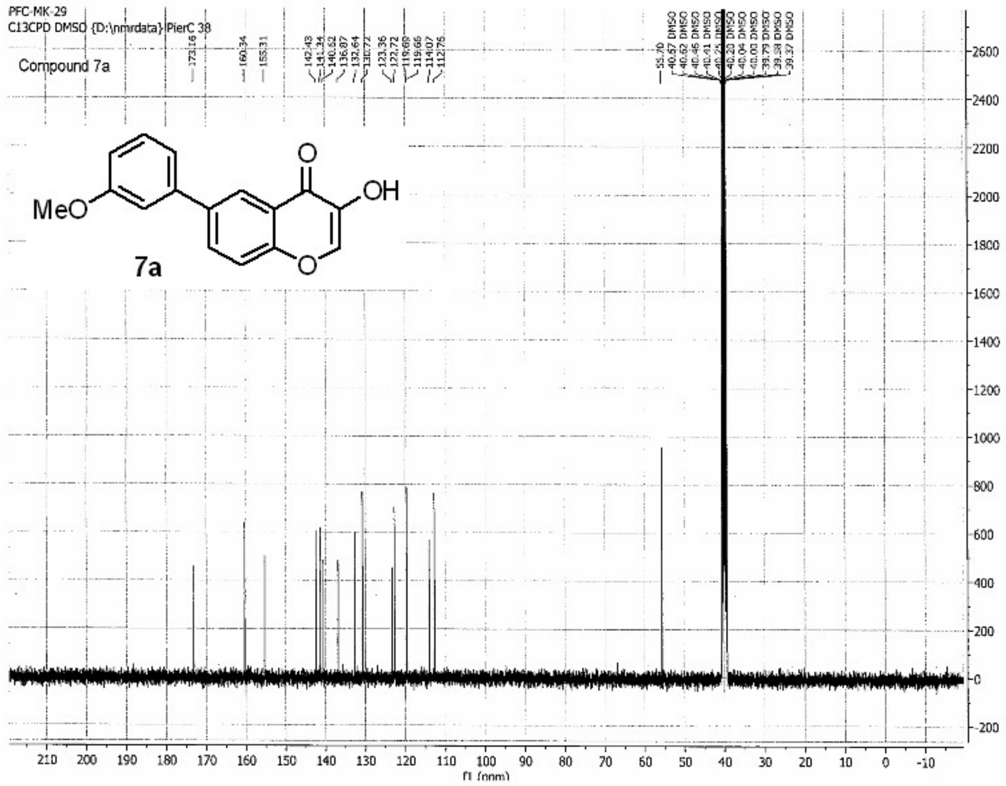

PFC-MK-29
C13CPD DMSO {D:\nmrdata} PierC 38
Compound 7a
173.16
160.34
155.31
142.43
130.72
123.36
122.72
119.69
119.66
114.07
112.75
55.70
MeO
OH
O
7a
f1 (ppm)

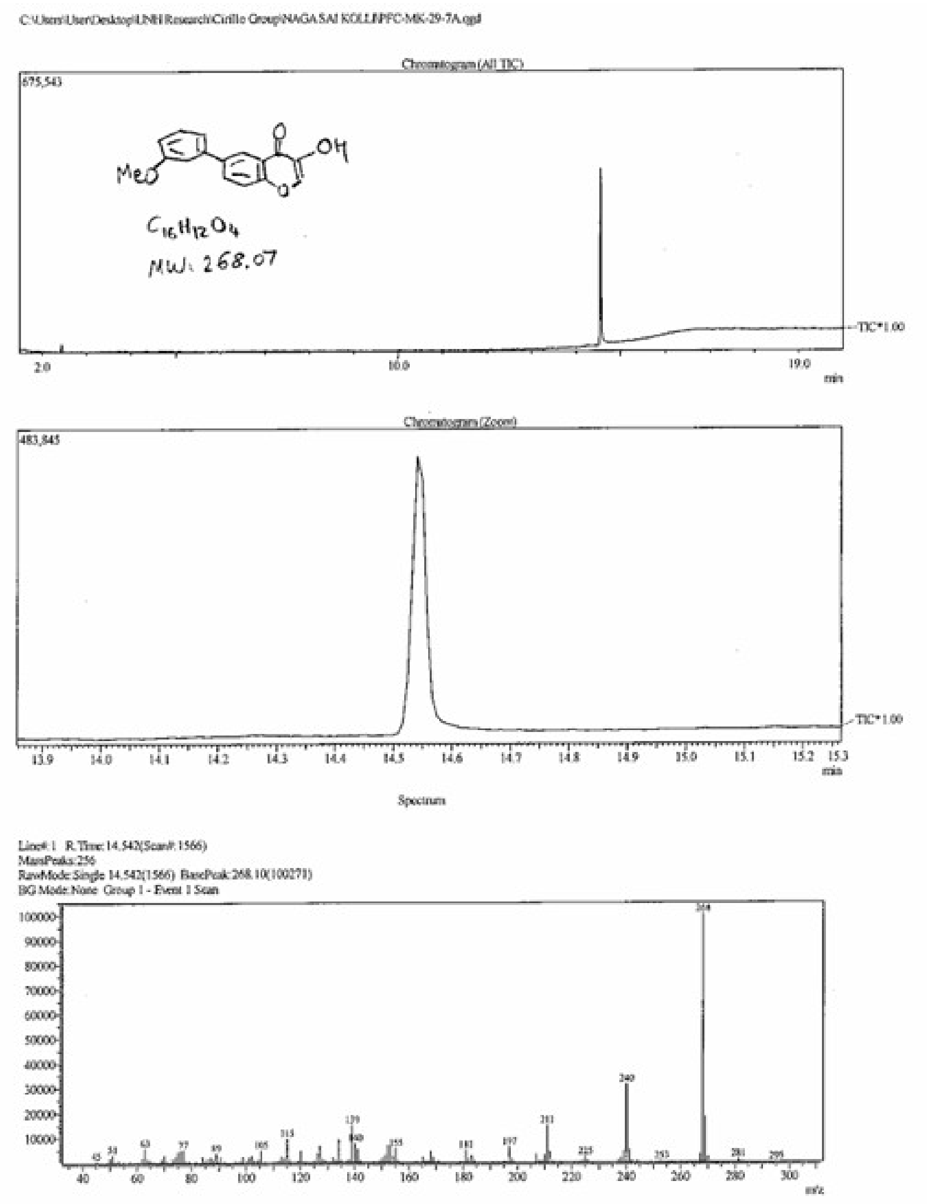

S1.7 6-(3-thiophenyl)-3-hydroxy-chromen-4-one, 7b.

Bromo-chromenone **6** (240 mg; 1.00 mmol; 1.0 equiv.) was dissolved in 25 mL 1,4-dioxane. Water (12 mL) was added, then 3-thienylboronic acid (154 mg; 1.20 mmol; 1.2 equiv.) and potassium carbonate (276 mg; 2.00 mmol; 2.0 equiv.). The mixture was purged with argon gas for 10 minutes, then $Pd(PPh_3)_2Cl_2$ catalyst (36 mg; 0.05 mmol; 0.05 equiv.) was added. The mixture was heated at 120 ^{o}C for 2 hours, during which time it turned dark brown, then it was allowed to cool and was quenched with water and extracted with ethyl acetate. The combined organic layers were washed with brine, dried with Na_2SO_4, decanted, and the solvent was removed *in vacuo*. The product was purified by column chromatography then triturated with dichloromethane to afford a yellow powder (153 mg; 63% yield). ^{1}H NMR (400 MHz;

$SO(CD_3)_2$) δ (ppm): 9.21 (br s, 1 H, OH); 8.33 (d, 1 H, J = 2.2 Hz); 8.26 (s, 1 H); 8.13 (dd, 1 H, J = 9.7 and 2.3 Hz); 8.05 (dd, 1 H, J = 2.8 and 1.4 Hz); 7.71-7.65 (m, 3 H). GC-MS: m/z = 244.

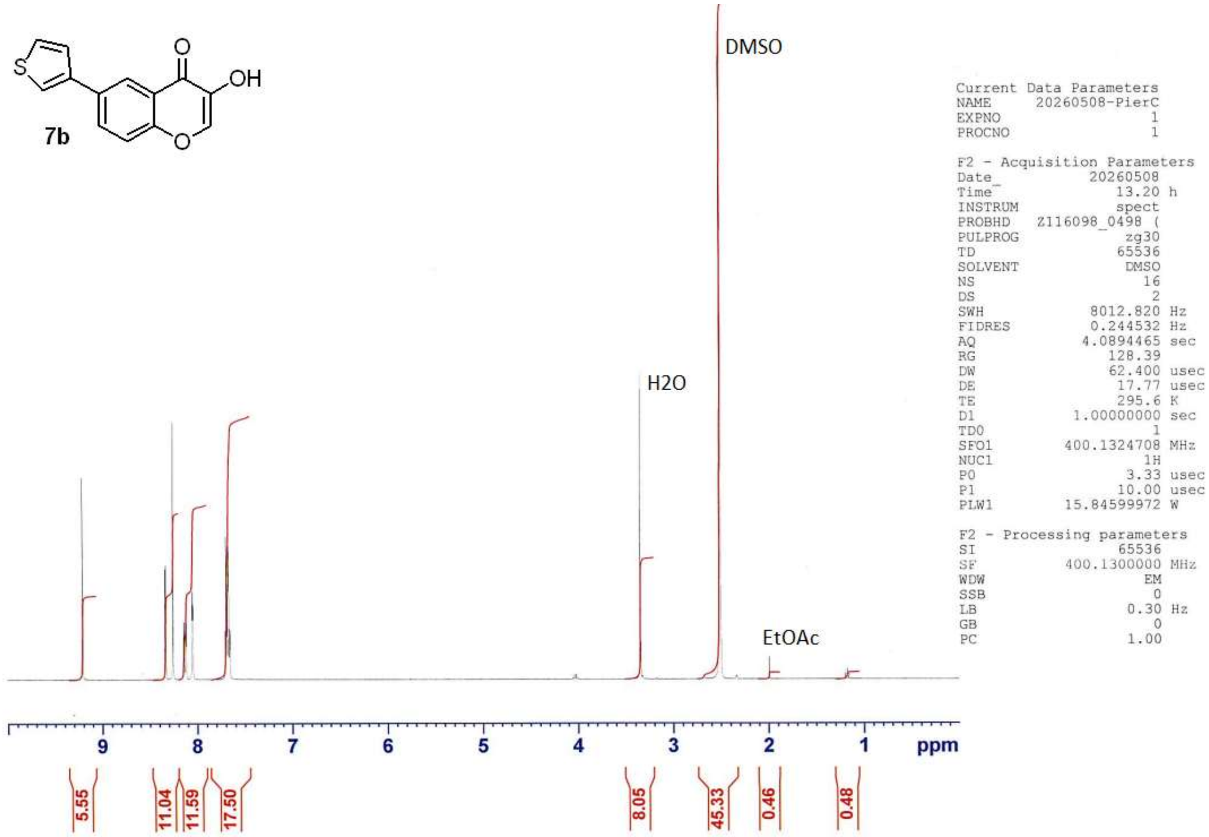

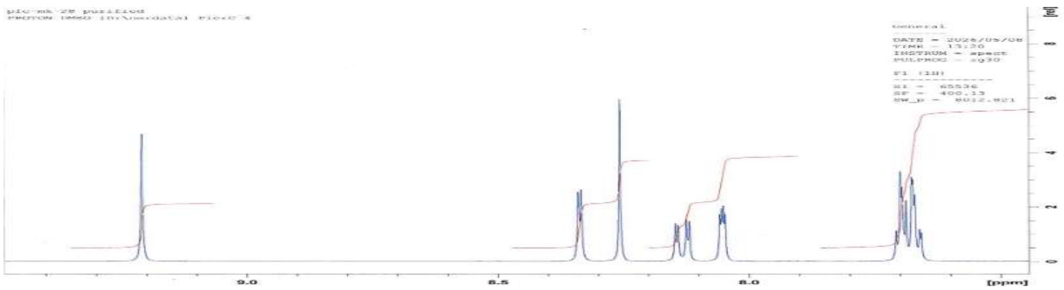

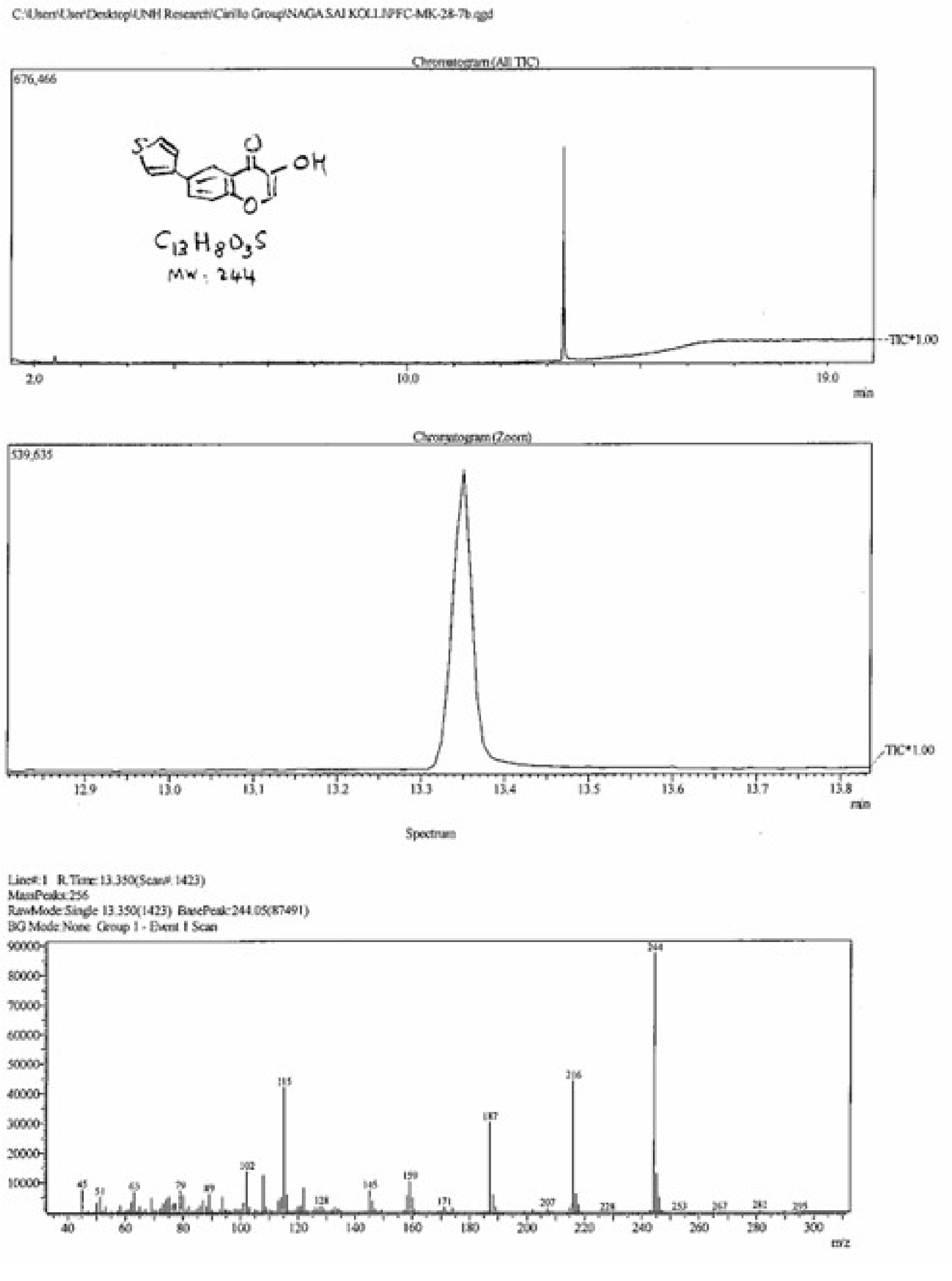

S2. Structural confirmation and molecular information.

S2.1 NSK 3. 4-Fluorobenzaldehyde (66 mg; 0.537 mmol; 1.2 equiv.) and 2-aminopyridine (51 mg; 53.7 mmol; 1.2 equiv.) were added to a 5 mL pear-shaped flask. Isopropanol (1.5 mL) was added, then once dissolution is completed, compound **7a** (120 mg; 0.447 mmol; 1.0 equiv.) was added. The resulting

suspension was heated to 85 °C (oil bath) for 1 hour. When the reaction was completed as monitored by TLC, the mixture was removed from the heat, allowed to cool, then ether was added and the pure product precipitated as a tan solid, which was collected by vacuum filtration using a Hirsch funnel (38 mg; 31% yield). ^{1}H NMR (400 MHz; $SO(CD_3)_2$) δ (ppm): 9.43 (s, 1 H, OH); 8.27 (s, 1 H); 8.07 (d, 1 H); 7.98 (d, 1 H); 7.65 (d, 1 H); 7.62-7.50 (m overlap, 3 H); 7.50-7.40 (m overlap, 2 H); 7.30 (d, 1 H); 7.27-7.16 (m overlap, 3 H); 6.99 (d, 1 H); 6.85-6.78 (m overlap, 2 H); 6.55 (dd, 1 H); 3.81 (s, 3 H, OCH_3). ^{13}C NMR (100 MHz; $SO(CD_3)_2$) δ (ppm): 172.91; 160.35; 157.88; 154.57; 151.49; 147.82; 140.55; 138.37; 137.44; 136.93; 136.53; 136.50; 132.67; 130.72; 129.45; 129.37; 122.69; 122.64; 119.66; 119.46; 115.91; 115.70; 114.10; 113.33; 112.71; 109.76; 55.71; 50.67.

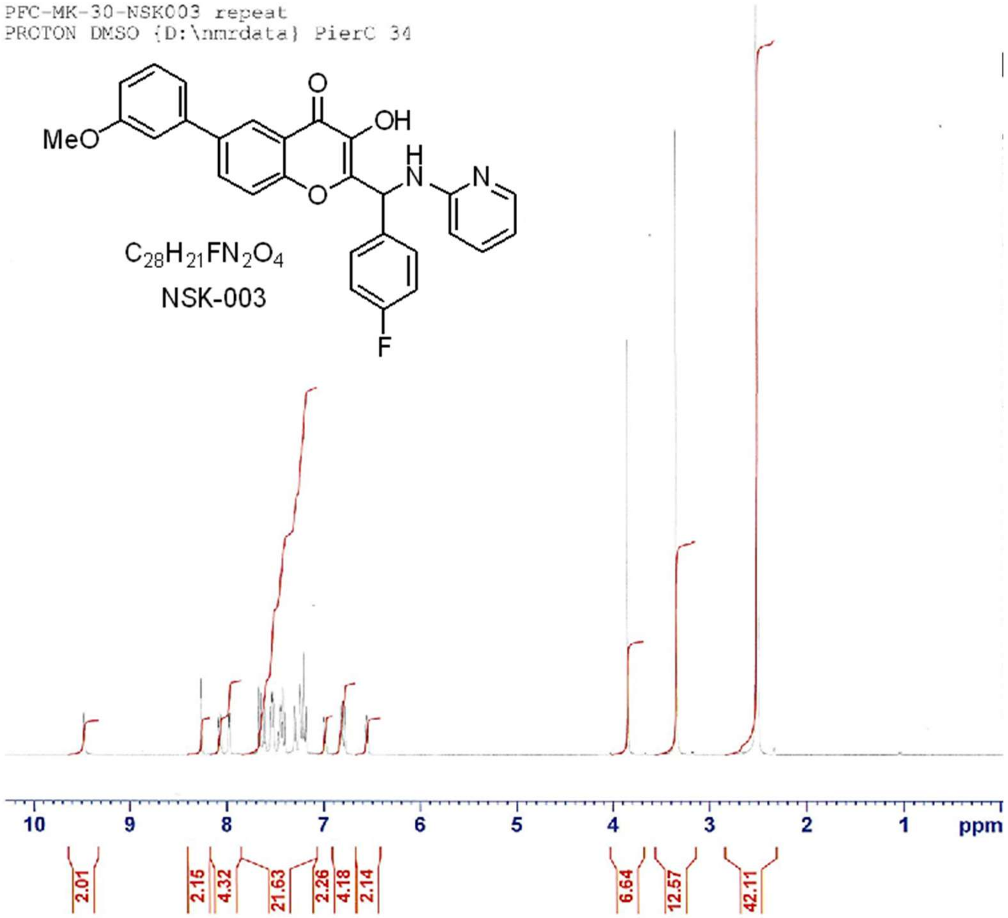

See expansion of region δ 9.4 to 6.6 ppm below (next page)

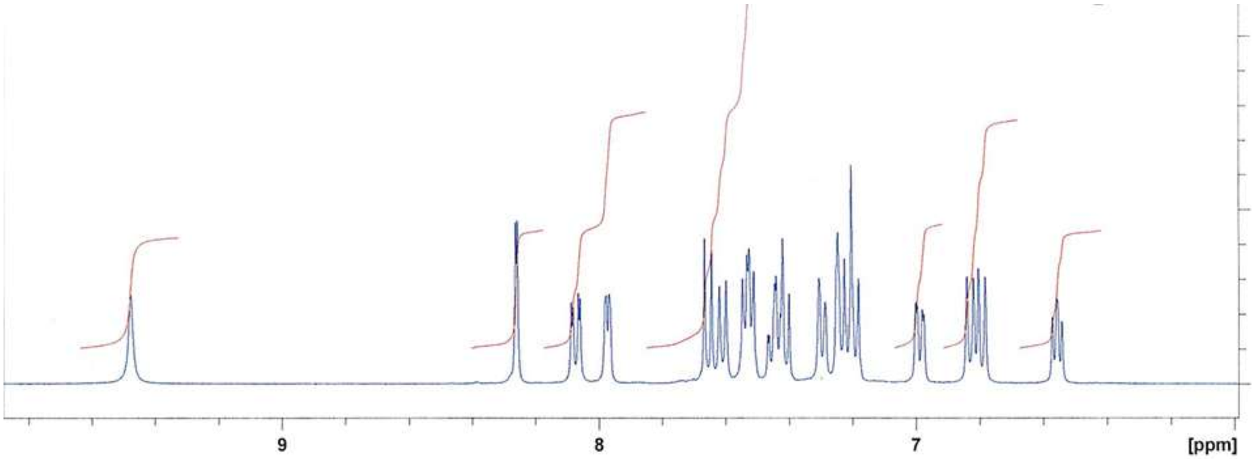
9
8
7
[ppm]

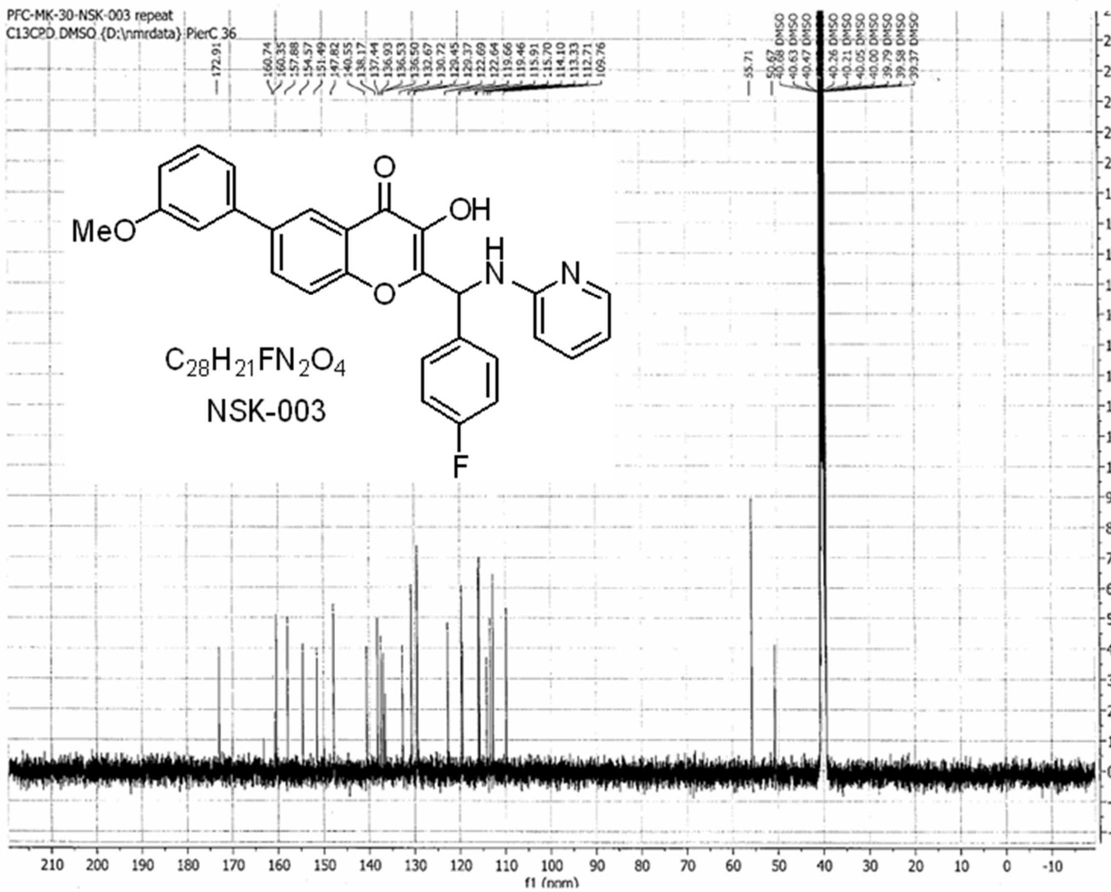
PFC-MK-30-NSK-003 repeat
C13CPD DMSO {D:\nmrdata} PierC 36
172.91
160.74
160.35
157.88
154.57
151.49
147.82
140.55
138.17
137.44
136.93
136.53
136.50
132.67
130.72
129.45
129.37
122.69
122.64
119.66
119.46
115.91
115.70
114.10
113.33
112.71
109.76
55.71
50.67
40.68 DMSO
40.63 DMSO
40.47 DMSO
40.26 DMSO
40.21 DMSO
40.05 DMSO
40.00 DMSO
39.79 DMSO
39.58 DMSO
39.37 DMSO
MeO
O
OH
H
N
N
O
F
$C_{28}H_{21}FN_2O_4$
NSK-003
f1 (ppm)

S2.2 NSK 4. Obtained from compound **7a**, 2-aminopyridine, and 4-trifluoromethylbenzaldehyde, following a similar procedure as described for NSK 3. ^{1}H NMR (400 MHz; $SO(CD_3)_2$) δ (ppm): 9.57 (s, 1 H, OH); 8.27 (s, 1 H); 8.08 (d, 1 H); 7.99 (s, 1 H); 7.80-7.63 (m overlap, 5 H); 7.50-7.41 (m overlap, 2 H); 7.31 (d, 1 H); 7.25 (s, 1 H); 7.00 (d, 1 H); 6.96 (d, 1 H); 6.81 (d, 1 H); 6.59 (dd, 1 H); 3.84 (s, 3 H, OCH_3).

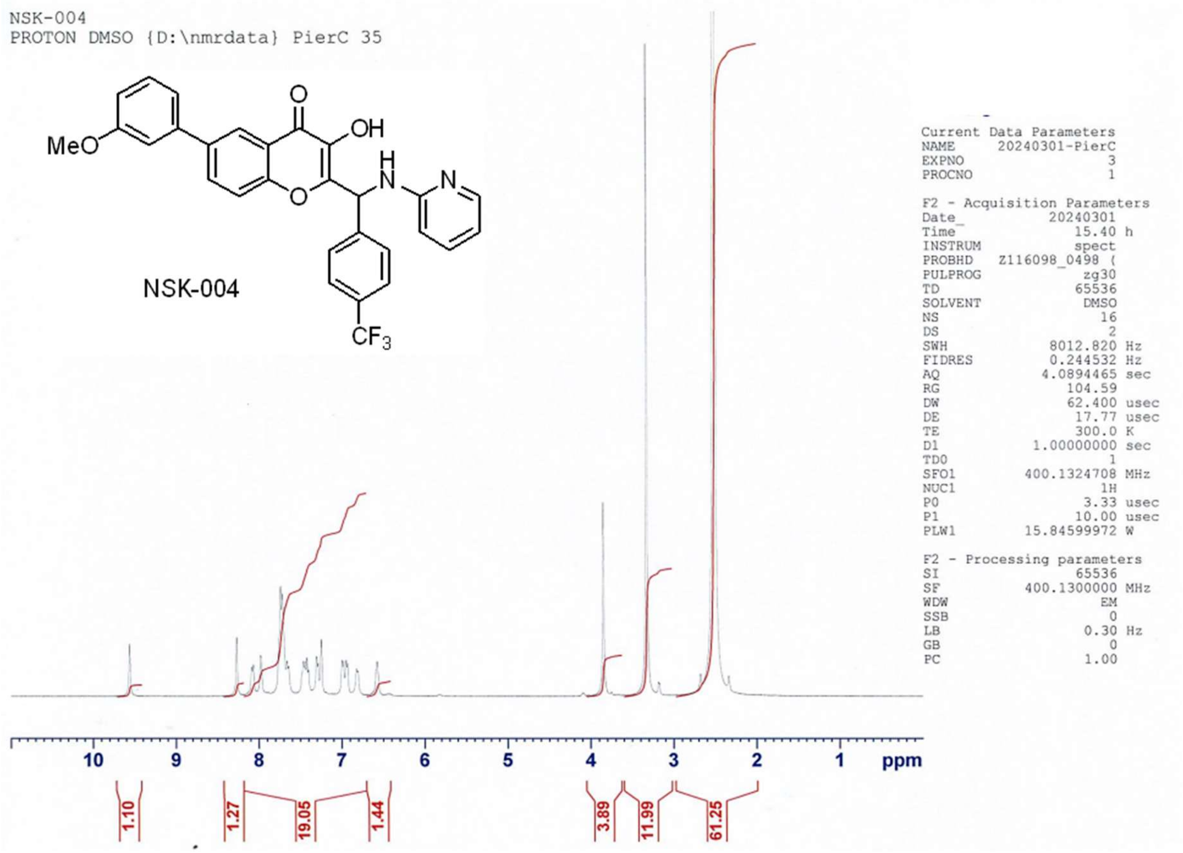

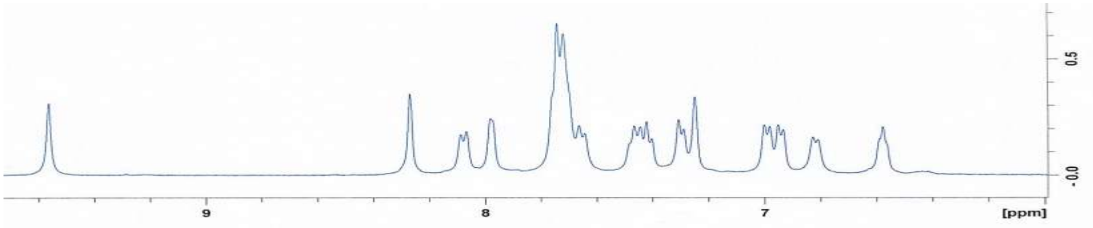

S2.3 NSK 6.

Obtained from compound **7b**, 2-aminopyridine, and 4-fluorobenzaldehyde, following a similar procedure as described for NSK 3. ^{1}H NMR (400 MHz; $SO(CD_3)_2$) δ (ppm): 9.41 (s, 1 H, OH); 8.30 (s, 1 H); 8.12 (d, 1 H); 8.04 (s, 1 H); 7.97 (d, 1 H); 7.52-7.70 (overlapping m, 6 H); 7.49 (dd, 1 H); 7.19 (dd, 2 H); 6.80 (dd, 2 H); 6.55 (dd, 1 H).

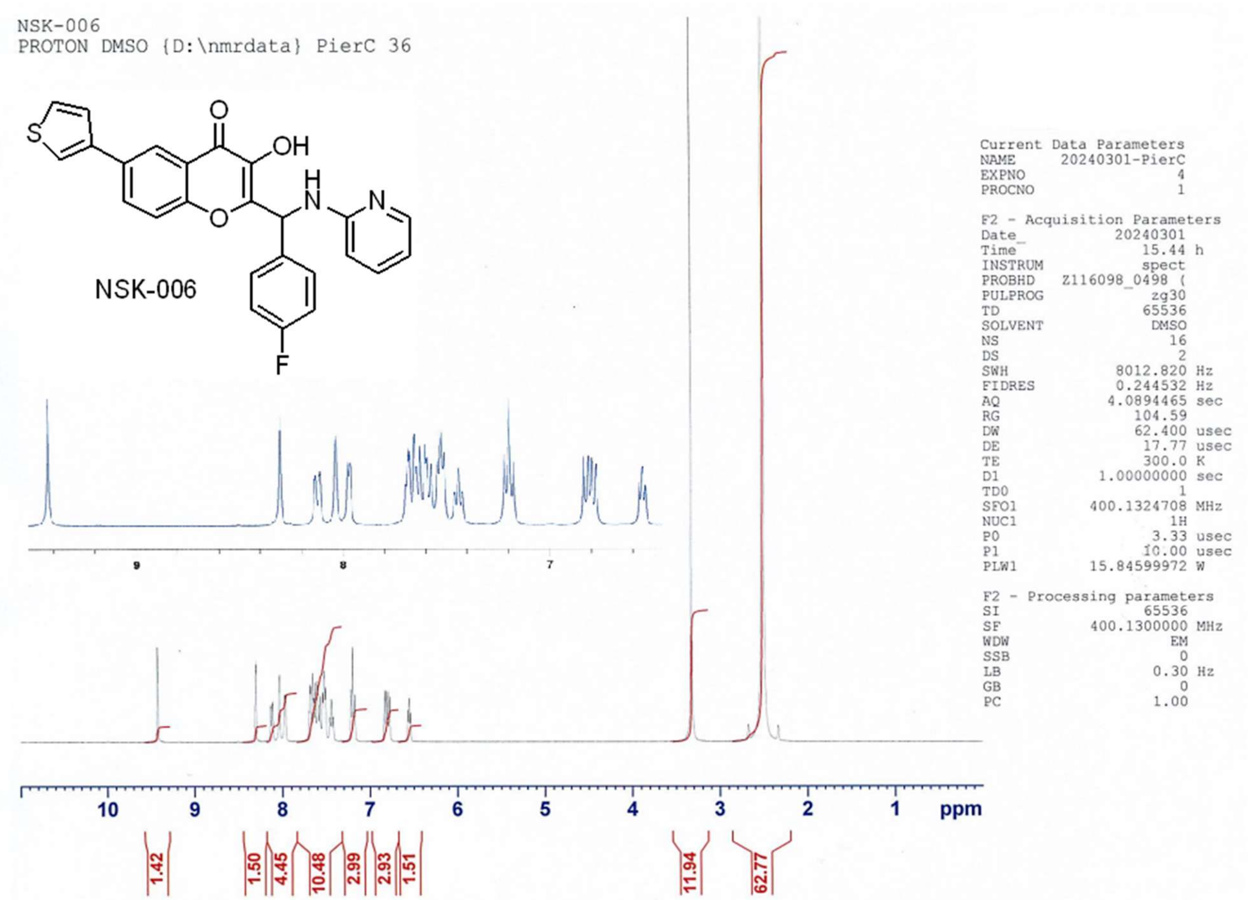

S2.4 NSK 7.

Obtained from compound **7b**, 2-aminopyridine, and 3-thiophene carbaldehyde, following a similar procedure as described for NSK 3. ^{1}H NMR (400 MHz; $SO(CD_3)_2$) δ (ppm): 9.40 (s, 1 H, OH); 8.30 (s, 1 H); 8.12 (d, 1 H, J = 7.9 Hz); 8.03 (s, 1 H); 7.99 (d, 1 H, J = 4.0 Hz); 7.68-7.60 (m overlap, 3 H); 7.58-7.50 (m overlap, 2 H); 7.43 (dd, 1 H, J = 7.2 and 7.4 Hz); 7.21 (d, 1 H, J = 4.6 Hz); 6.85 (d, 1 H, J = 8.4 Hz); 6.77 (d, 1 H, J = 8.3 Hz); 6.55 (dd, 1 H, J = 5.7 and 5.8 Hz).

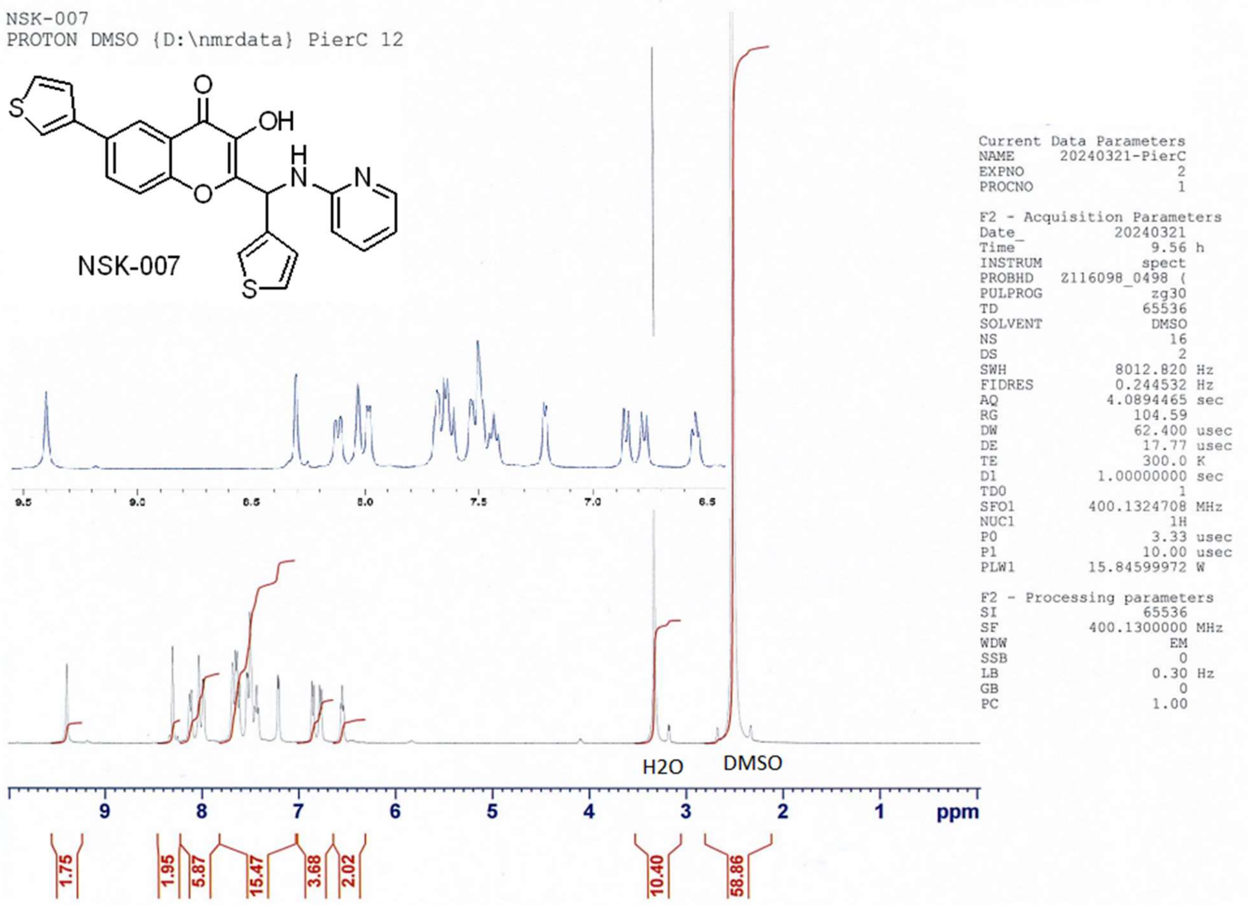

S2.5 NSK 12. Obtained from compound **7a**, 2-aminopyridine, and benzaldehyde, following a similar procedure as described for NSK 3. A light tan solid; 69% yield. ^{1}H NMR (400 MHz; SO(CD$_3$)$_2$) δ (ppm): 9.42 (br s, 1 H, OH); 8.26 (d, 1 H, J = 2.0 Hz); 8.06 (d, 1 H, J = 7.1 Hz); 7.96 (d, 1 H, J = 4.7 Hz); 7.65 (d, 1 H, J = 8.8 Hz); 7.58 (d, 1 H, J = 7.4 Hz); 7.50 (d, 1 H, J = 7.4 Hz); 7.45-7.33 (m overlap, 6 H); 7.29 (d, 1 H, J = 6.2 Hz); 7.24 (s, 1 H); 6.98 (dd, 1 H, J = 8.0 and 1.6 Hz); 6.85 (d, 1 H, J = 8.3 Hz); 6.80 (d, 1 H, J = 8.2 Hz); 6.55 (dd, 1 H, J = 6.0 and 6.4 Hz); 3.83 (s, 3 H, OCH$_3$). ^{13}C NMR (100 MHz; SO(CD$_3$)$_2$) δ (ppm): 172.87; 160.34; 158.00; 154,56; 151.82; 147.80; 140.56; 140.33; 138.21; 137.40; 136.90; 132.63; 130.72; 129.03; 127.92; 127.43; 122.69; 122.64; 119.65; 119.45; 114.09; 113.22; 112.69; 109.74; 55.70; 51.24.

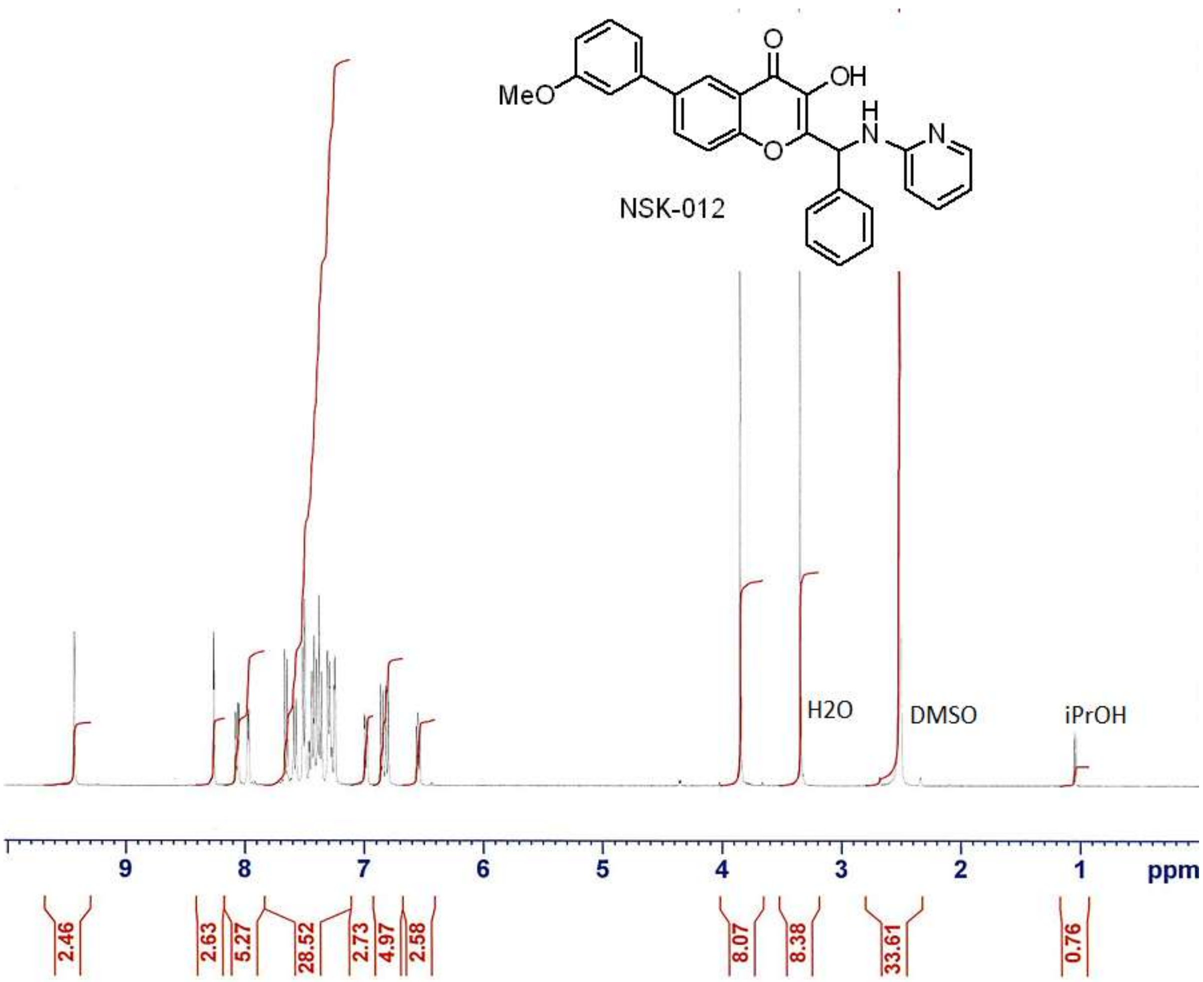

See expansion of region δ 9.5 to 6.5 ppm below (next page)

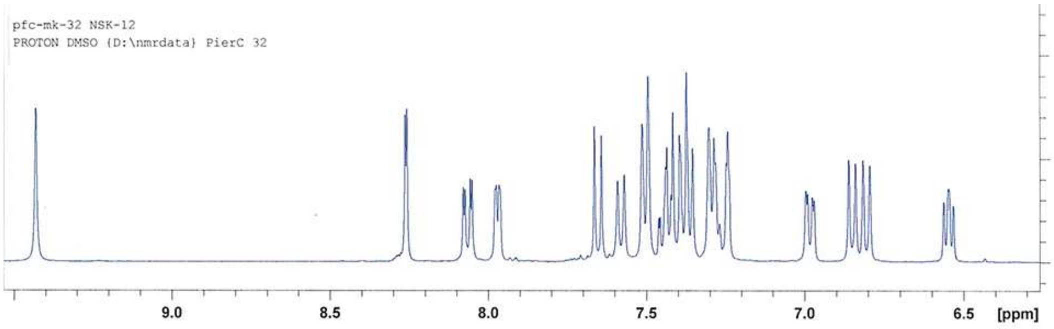

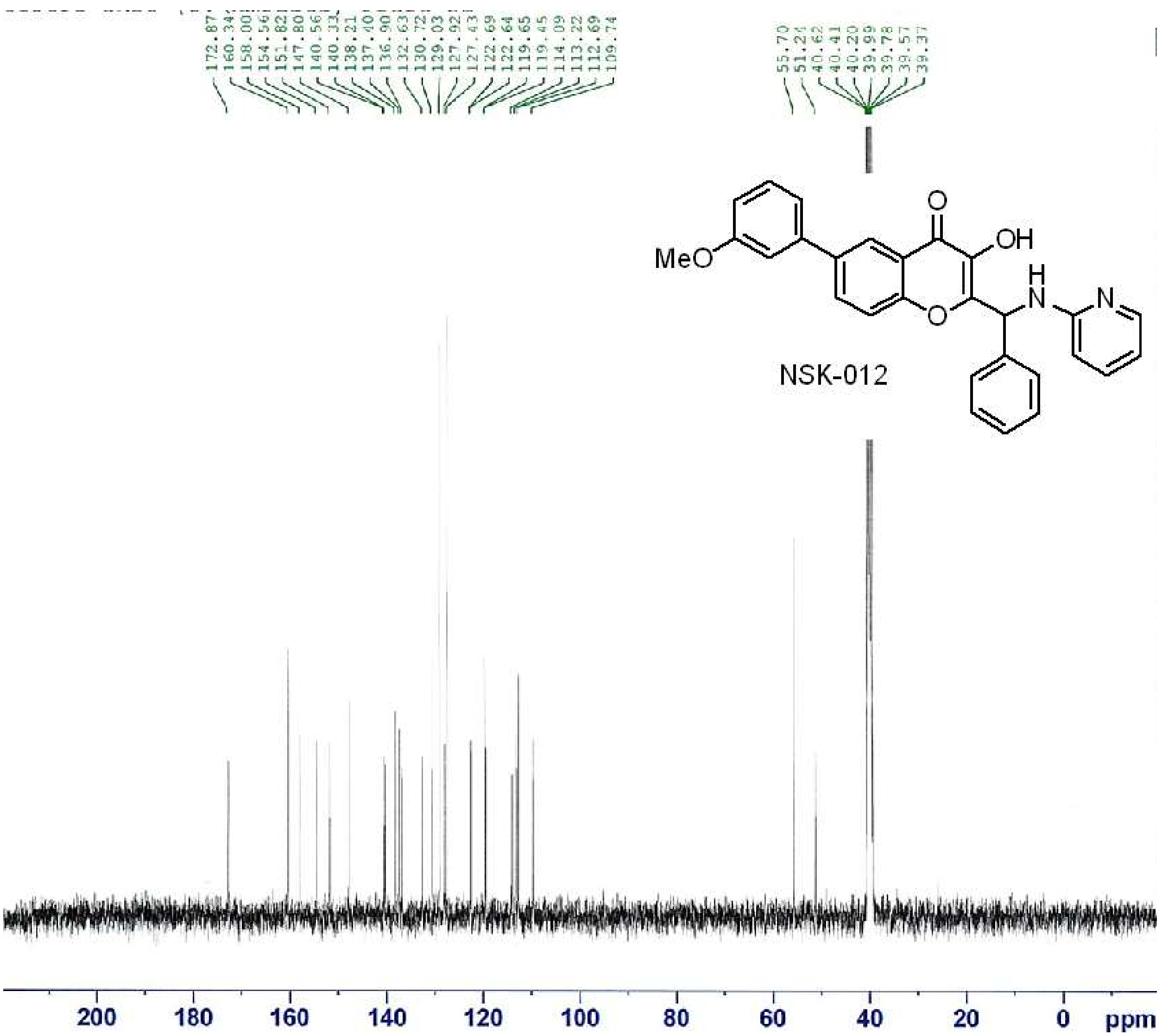

S2.6 NSK 14.

Obtained from compound **6**, 2-aminopyridine, and benzaldehyde, following a similar procedure as described for NSK 3. An off-white solid; 83% yield. ^{1}H NMR (400 MHz; $SO(CD_3)_2$) δ (ppm): 9.56 (br s, 1 H, OH); 8.15 (d, 1 H, J = 2.0 Hz); 7.94 (d, 1 H, J = 5.0 Hz); 7.88 (dd, 1 H, J = 10.0 and 2.0 Hz); 7.56-7.51 (m overlap, 2 H), 7.49-7.46 (m overlap, 2 H); 7.44 (dd, 1 H, J = 10.0 and 8.5 Hz); 7.38-7.32 (m, 2 H); 7.28 (d, 1 H, J = 6.5 Hz); 6.82 (d, 1 H, J = 8.8 Hz); 6.77 (d, 1 H, J = 8.8 Hz); 6.53 (dd, 1 H, J = 7.5 and 7.5 Hz).

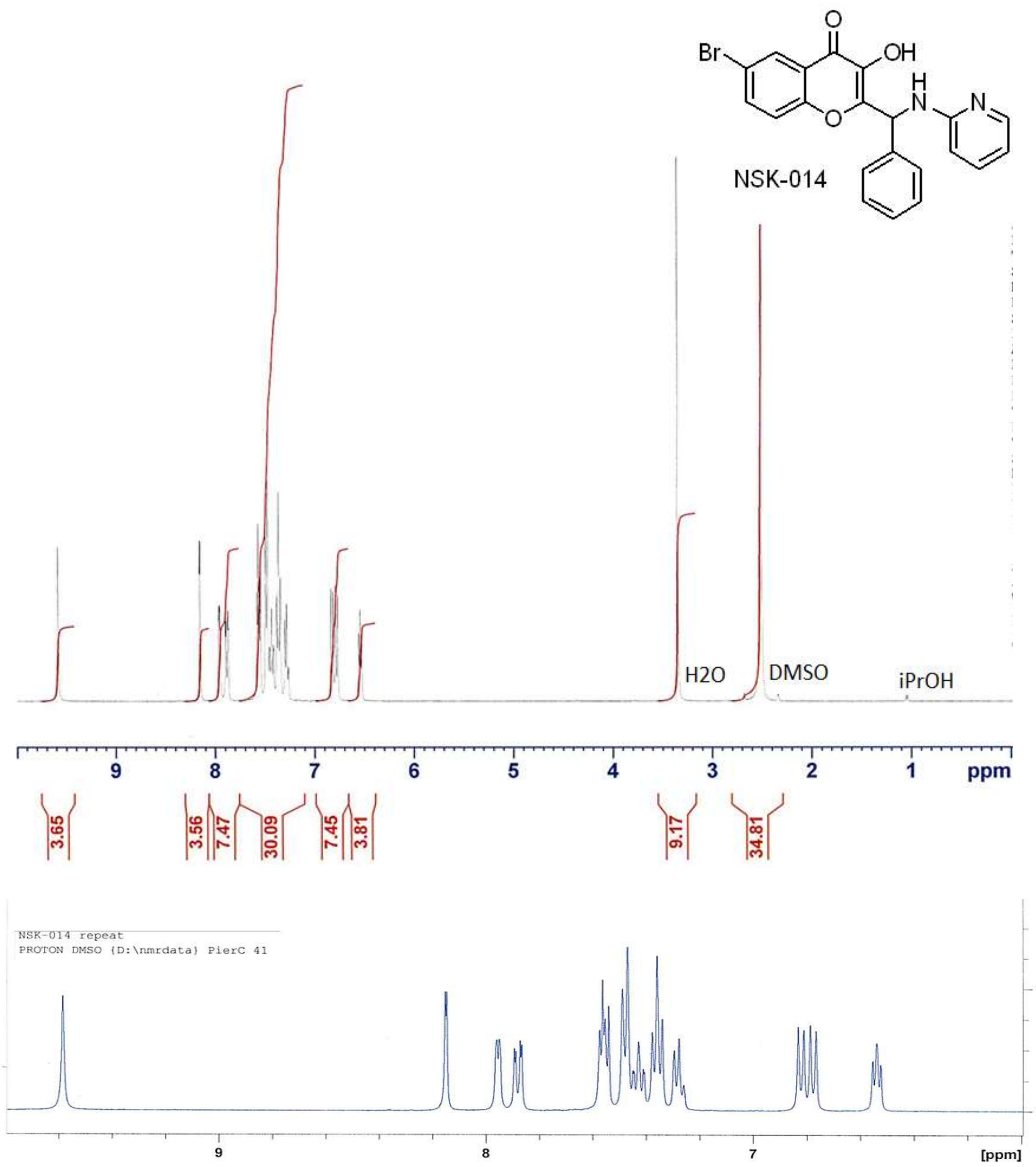

S2.7 NSK 15.

Obtained from compound **6**, 2-aminopyridine, and benzaldehyde, following a similar procedure as described for NSK 3. ^{1}H NMR (400 MHz; $SO(CD_3)_2$) δ (ppm): 9.70 (s, 1 H); 8.18 (s, 1 H); 7.99 (s, 1 H); 7.89 (d, 1 H); 7.75-7.60 (m overlap, 5 H); 7.51 (d, 1 H); 7.42 (dd, 1 H); 6.91 (d, 1 H); 6.80 (d, 1 H); 6.50 (dd, 1 H).

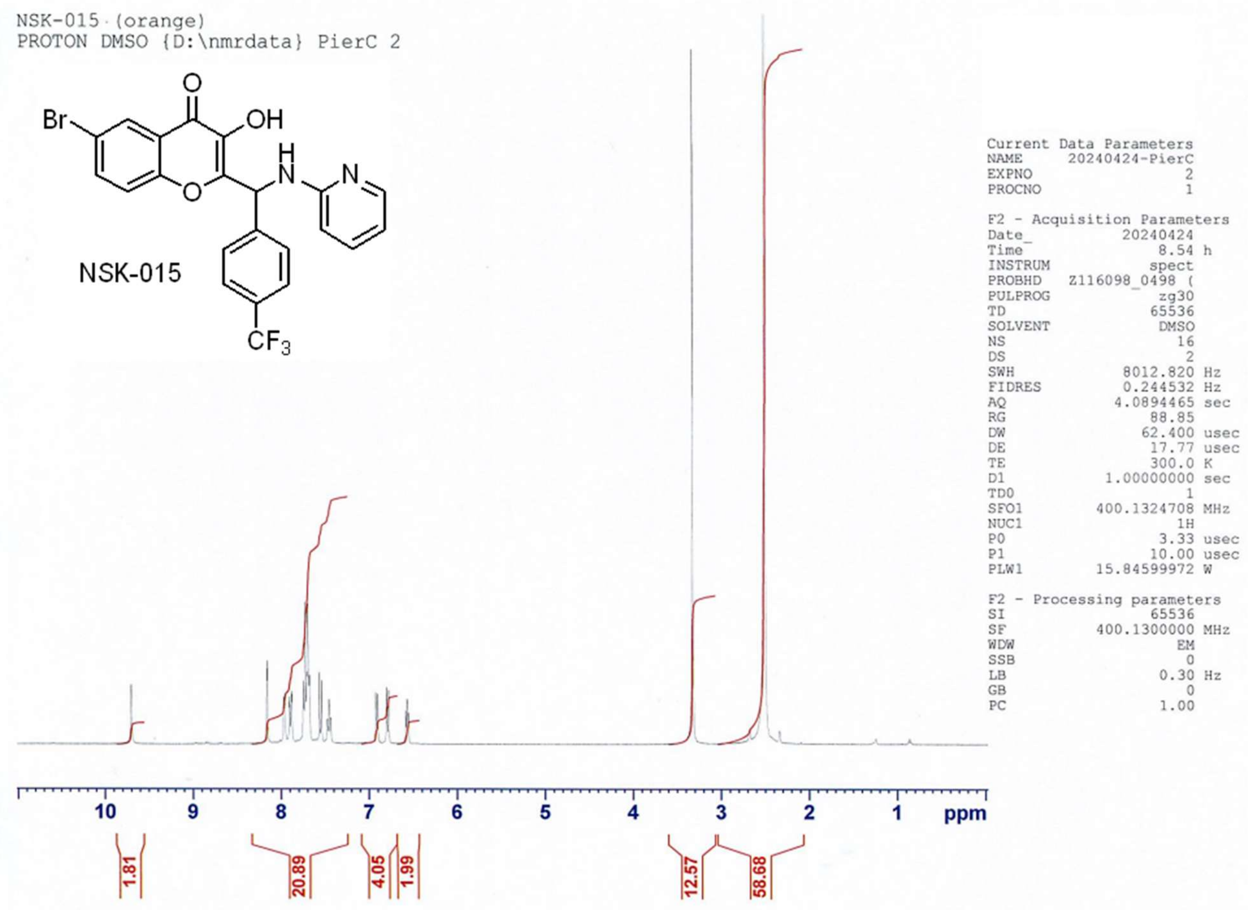

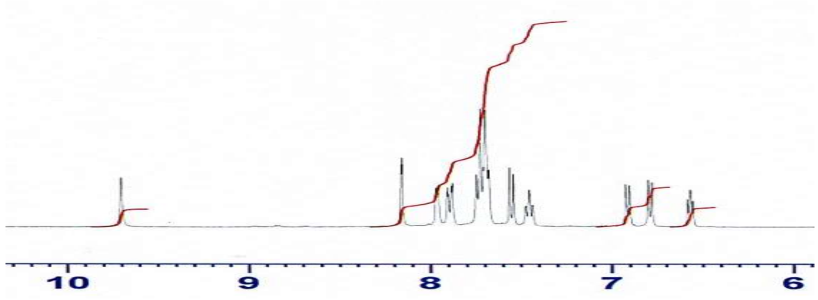

S2.8 NSK 16.

Obtained from compound **6**, 2-aminopyridine, and benzaldehyde, following a similar procedure as described for NSK 3. A light tan powder; 94% yield. ^{1}H NMR (400 MHz; $SO(CD_3)_2$) δ (ppm): 9.55 (s, 1 H); 8.16 (s, 1 H); 8.00 (s, 1 H); 7.90 (d, 1 H); 7.50-7.30 (m overlap, 5 H); 7.19 (d, 1 H); 6.81 (d, 1 H); 6.75 (d, 1 H); 6.53 (m, 1 H). ^{13}C NMR (100 MHz; $SO(CD_3)_2$) δ (ppm): 171.75; 157.80; 153.88; 152.19; 147.73; 140.42; 138.19; 137.47; 136.47; 127.50; 127.38; 127.19; 124.18; 123.05; 121.40; 117.37; 113.28; 109.71; 47.89.

NSK-016
PROTON DMSO {D:\nmrdata} PierC 3

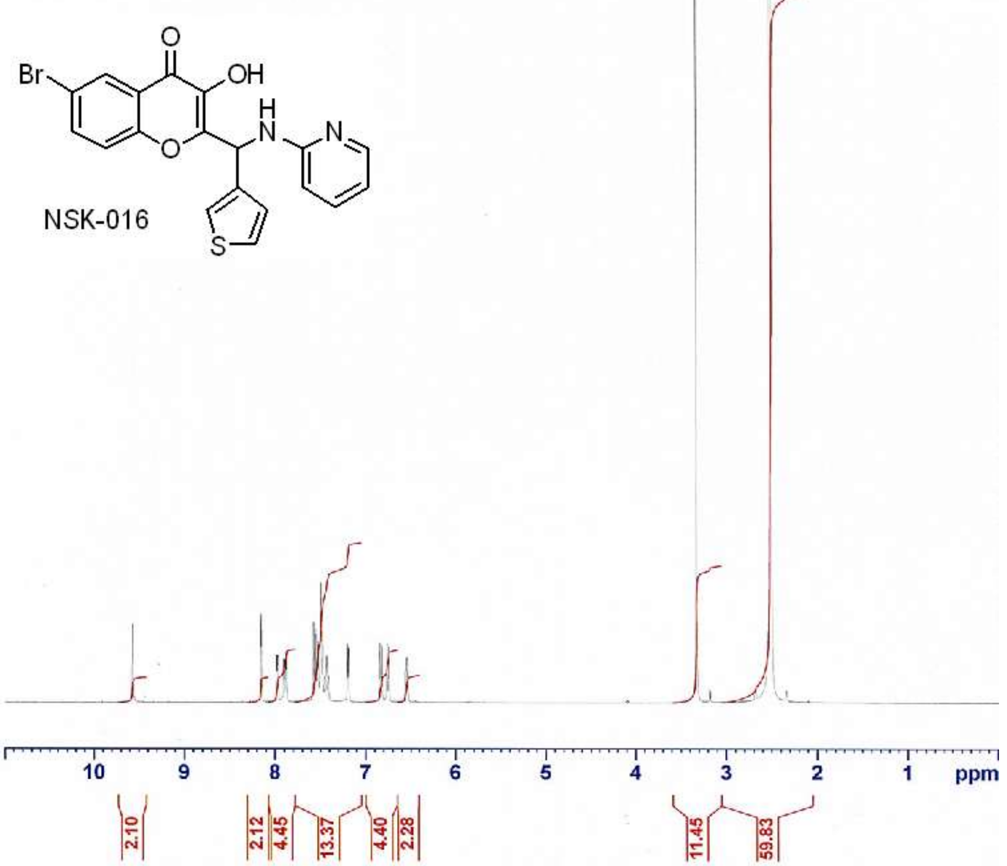

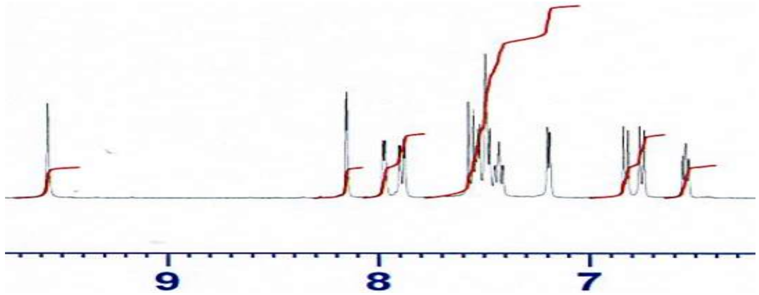

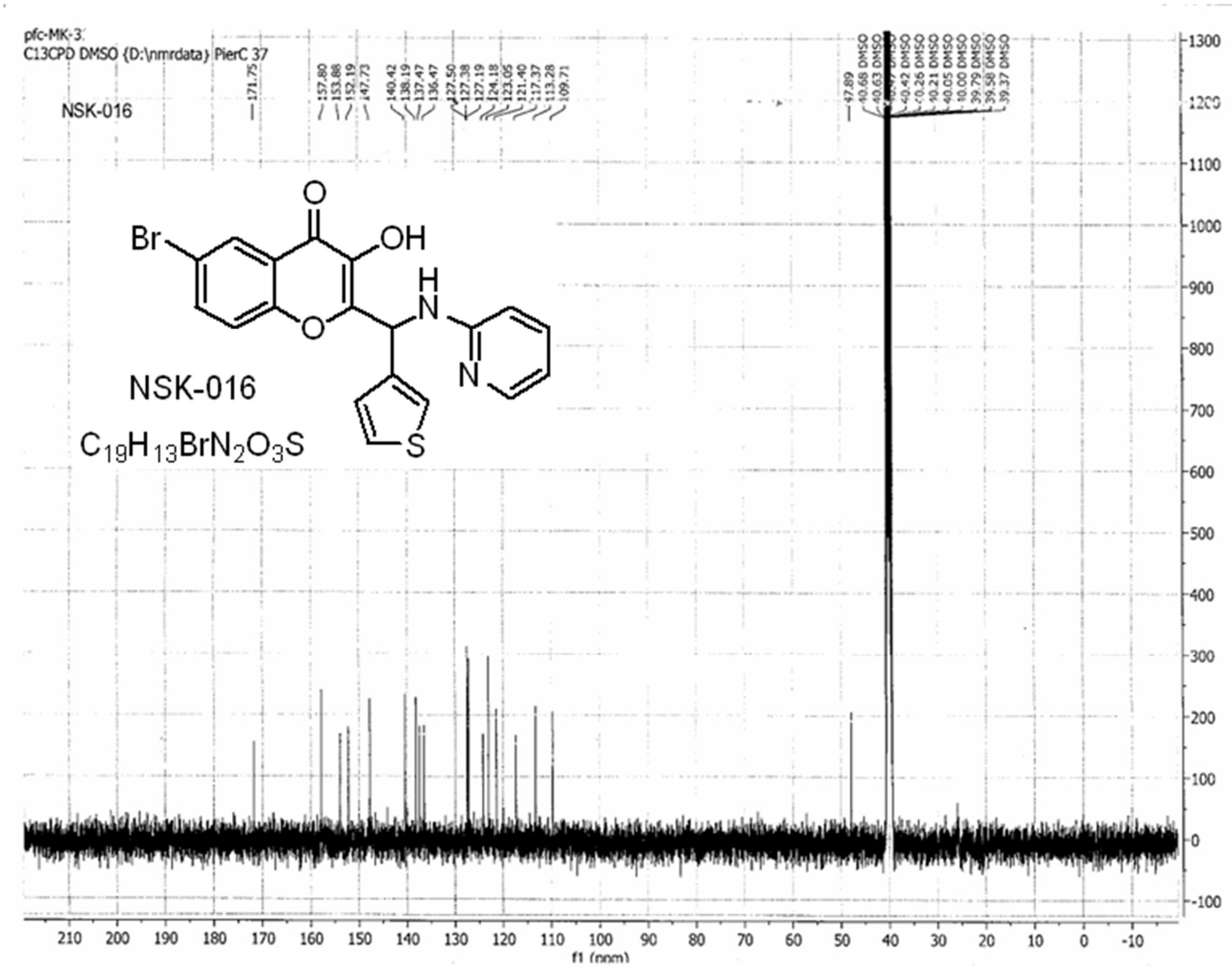

pfc-MK-3
C13CPD DMSO {D:\nmrdata} PierC 37
NSK-016
171.75
157.80
153.88
152.19
147.73
140.42
138.19
137.47
136.47
127.50
127.38
127.19
124.18
123.05
121.40
117.37
113.28
109.71
47.89
Br
O
OH
H
N
N
S
NSK-016
C19H13BrN2O3S
f1 (ppm)

S2.9 NSK 18.

Obtained from compound **6**, 2-aminopyridine, and 3-fluorobenzaldehyde, following a similar procedure as described for NSK 3. ^{1}H NMR (400 MHz; $SO(CD_3)_2$) δ (ppm): 9.62 (s, 1 H); 8.15 (s, 1 H); 7.98 (s, 1 H); 7.89 (d, 1 H); 7.61 (d, 1 H); 7.58 (d, 1 H); 7.50-7.40 (m overlap, 2 H); 7.31-7.25 (m overlap, 2 H); 7.10 (dd, 1 H); 6.85 (d, 1 H); 6.78 (d, 1 H); 6.55 (dd, 1 H).

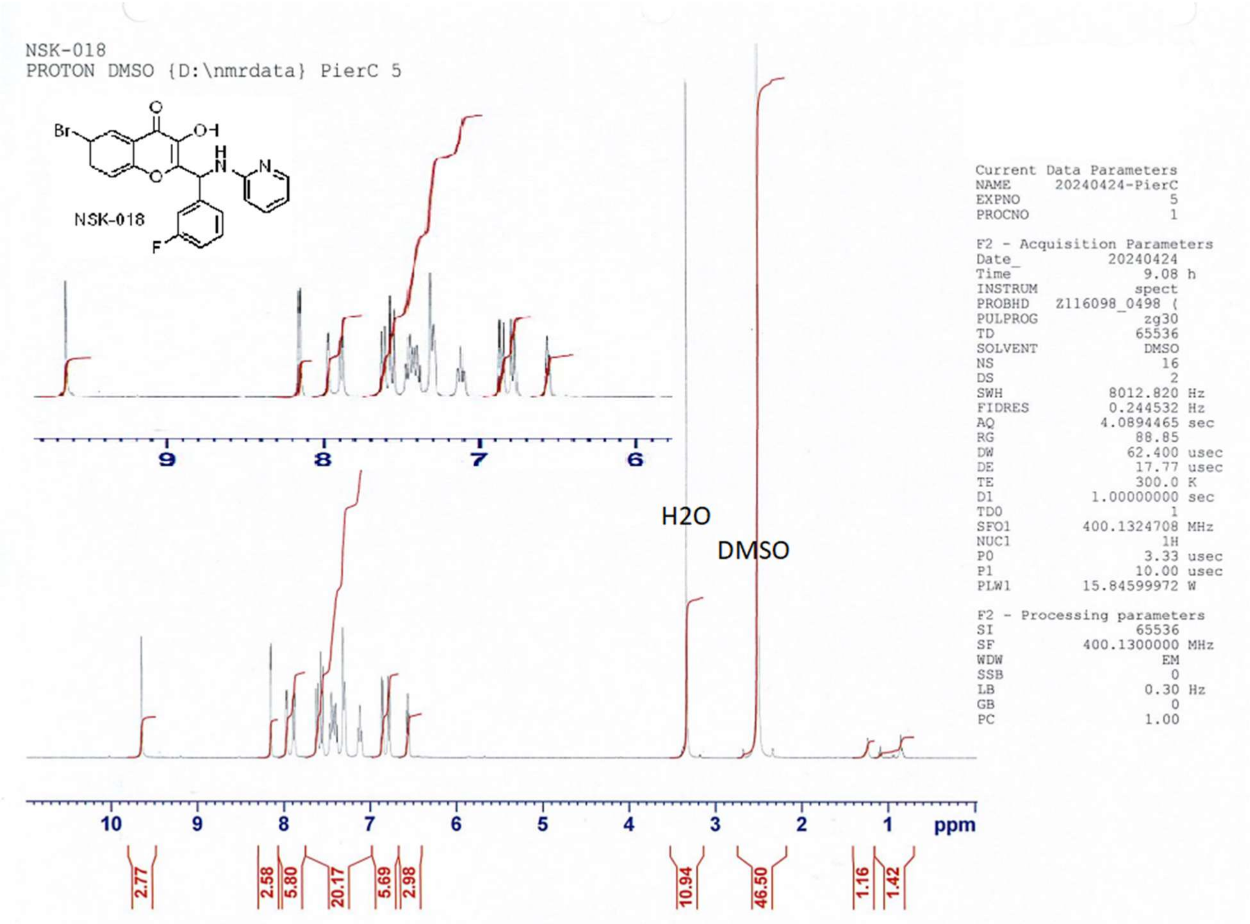

S**2.10 NSK 19**.

Obtained from compound **6**, 2-aminopyridine, and 2-fluorobenzaldehyde, following a similar procedure as described for NSK 3. ^{1}H NMR (400 MHz; SO(CD$_3$)$_2$) δ (ppm): 9.51 (s. 1 H); 8.18 (s, 1 H); 7.96 (s, 1 H); 7.89 (d, 1 H); 7.65-7.52 (overlap m, 3 H); 7.46 (dd, 1 H); 7.39 (dd, 1 H); 7.27-7.15 (m, 2 H); 6.99 (d, 1 H); 6.73 (d, 1 H); 6.56 (dd, 1 H).

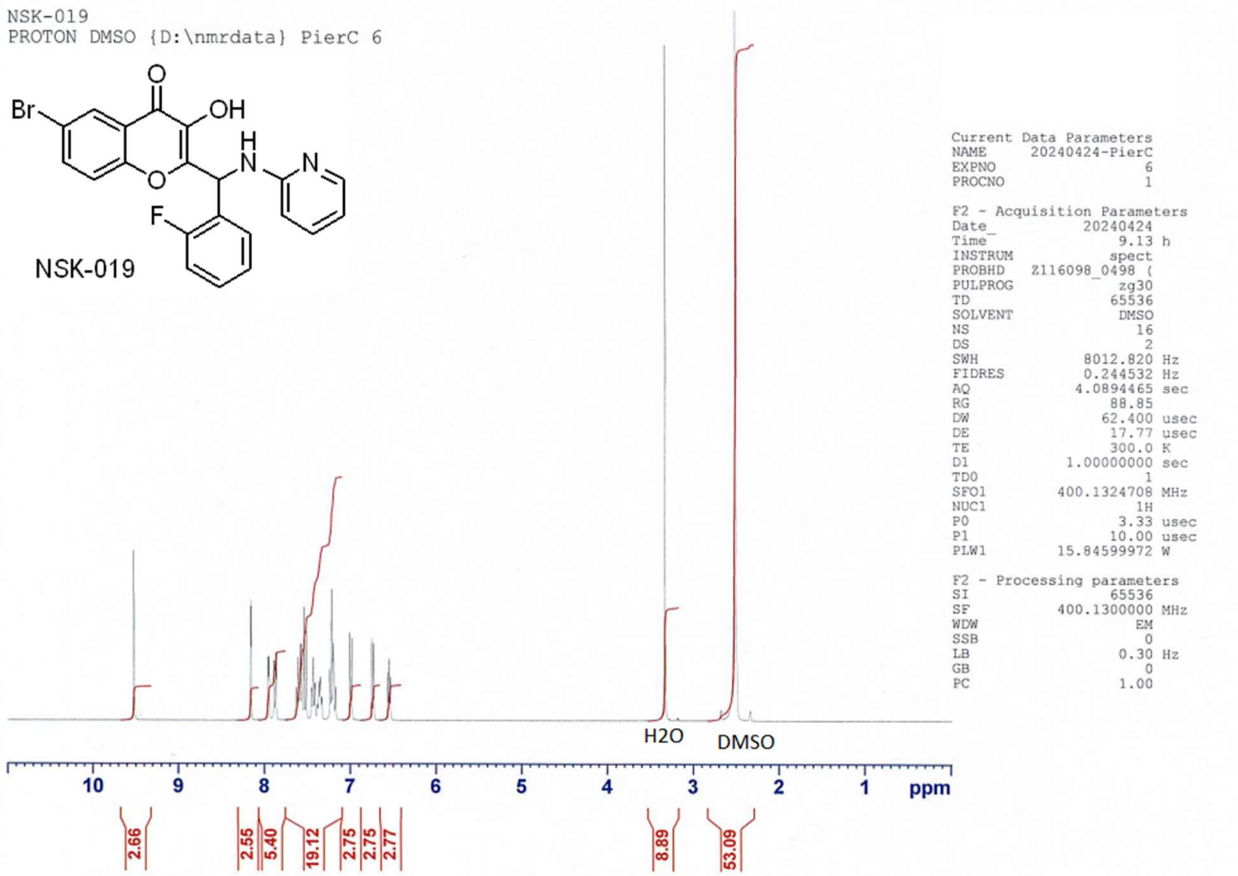

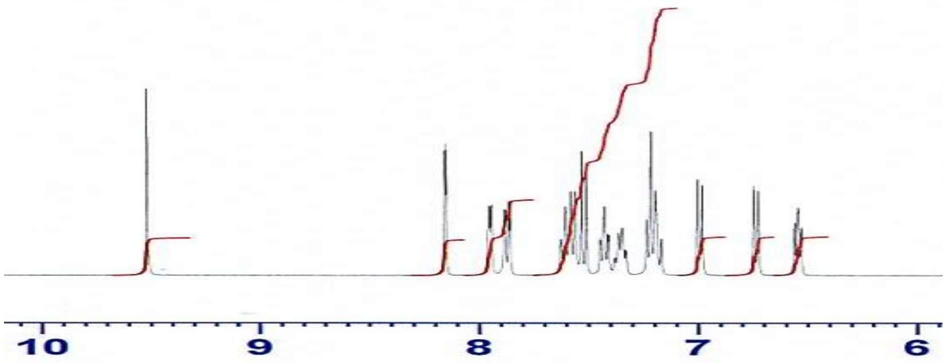

Table S1. Summary of compound concentrations, stock concentrations, and dilution series used for each microorganism and compound for growth-inhibition experiments.

<table>
<tr><th>Microorganism</th><th>Compounds</th><th>Compound Concentration in 250 µL Bacterial solutions (µg/mL)</th><th>Stock Concentrations in DMSO (mg/mL)</th><th>Volume of Stock Solutions added into 249.5 µL Bacterial Solutions for the Final Compound Concentrations (µL)</th></tr>
<tr><td rowspan="10">P. aeruginosa
PAO1
&
S. aureus
ALC2085</td><td>NSK 3</td><td rowspan="6">200,
100,
80,
40,
20,
10,
1</td><td rowspan="6">100,
50,
40,
20,
5,
0.5</td><td rowspan="11">0.5</td></tr>
<tr><td>NSK 4</td></tr>
<tr><td>NSK 6</td></tr>
<tr><td>NSK 7</td></tr>
<tr><td>NSK 12</td></tr>
<tr><td>NSK 14</td></tr>
<tr><td>NSK 15</td><td rowspan="5">40,
20,
10,
5,
2.5
1.25</td><td rowspan="5">20,
10,
5,
2.5,
1.25,
0.625</td></tr>
<tr><td>NSK 16</td></tr>
<tr><td>NSK 18</td></tr>
<tr><td>NSK 19</td></tr>
<tr><td>P. aeruginosa
PAO1</td><td>CIP</td></tr>
</table>
